# Genome-wide identification, and phylogenetic and expression profiling analyses, of *XTH* gene families in *Brassica rapa* L. and *Brassica oleracea* L.

**DOI:** 10.1186/s12864-020-07153-1

**Published:** 2020-11-11

**Authors:** Di Wu, Anqi Liu, Xiaoyu Qu, Jiayi Liang, Min Song

**Affiliations:** Qufu Normal University, College of Life Science, Qufu, 273165 P.R. China

**Keywords:** XTH, *Brassica rapa*, *Brassica oleracea*, Tissue expression; comparative genomics

## Abstract

**Background:**

Xyloglucan endotransglucosylase/hydrolase genes (*XTHs*) are a multigene family and play key roles in regulating cell wall extensibility in plant growth and development. *Brassica rapa* and *Brassica oleracea* contain XTHs, but detailed identification and characterization of the XTH family in these species, and analysis of their tissue expression profiles, have not previously been carried out.

**Results:**

In this study, 53 and 38 *XTH* genes were identified in *B. rapa* and *B. oleracea* respectively, which contained some novel members not observed in previous studies. All XTHs of *B. rapa*, *B. oleracea* and *Arabidopsis thaliana* could be classified into three groups, Group I/II, III and the Early diverging group, based on phylogenetic relationships. Gene structures and motif patterns were similar within each group. All XTHs in this study contained two characteristic conserved domains (Glyco_hydro and XET_C). XTHs are located mainly in the cell wall but some are also located in the cytoplasm. Analyses of the mechanisms of gene family expansion revealed that whole-genome triplication (WGT) events and tandem duplication (TD) may have been the major mechanisms accounting for the expansion of the *XTH* gene family. Interestingly, TD genes all belonged to Group I/II, suggesting that TD was the main reason for the largest number of genes being in these groups. *B. oleracea* had lost more of the *XTH* genes, the conserved domain XET_C and the conserved active-site motif EXDXE compared with *B. rapa*, consistent with asymmetrical evolution between the two *Brassica* genomes. A majority of *XTH* genes exhibited different tissue-specific expression patterns based on RNA-seq data analyses. Moreover, there was differential expression of duplicated *XTH* genes in the two species, indicating that their functional differentiation occurred after *B. rapa* and *B. oleracea* diverged from a common ancestor.

**Conclusions:**

We carried out the first systematic analysis of *XTH* gene families in *B. rapa* and *B. oleracea*. The results of this investigation can be used for reference in further studies on the functions of *XTH* genes and the evolution of this multigene family.

**Supplementary Information:**

**Supplementary information** accompanies this paper at 10.1186/s12864-020-07153-1.

## Background

The cell wall is an important characteristic structure in plant cells. Cell proliferation and volume increase are inseparable from the process of cell wall reconstruction. Xyloglucan is a component of hemicellulose in the primary cell wall of higher plants. It consists of a cellulose chain with side chains of oligosaccharides, each composed of a few xylose residues. Cell wall reconstruction is accompanied by breakage and regeneration of the cell wall xyloglucan. Xyloglucan endotransglucosylase/hydrolase (XTH) can catalyze the breakage and connection of xyloglucan molecules and modify the fiber-xyloglucan composite structure of plant cell walls, making it one of the key enzymes in cell wall remodeling [1, 2].

The XTH family belongs to the glycoside hydrolase family 16 (GH16); common features of proteins in this family are that they adopt a common β-jelly-roll fold and are active on a range of terrestrial and marine polysaccharides [3–5]. XTH generally performs two catalytic functions, one being xyloglucan endoglucosidase (XEH) activity and the other being xyloglucan endohydrolase (XET) activity, which specifically hydrolyzes xyloglucan glycosidic bonds and promotes cell wall expansion, degradation, repair and morphogenesis [6–8]. Based on the structural characteristics of XTH proteins, they can be divided into three groups, named I/II, III and the early diverging group. Of those reported to date, XTHs with glycosyltransferase activity belong mainly to Group I/II and those with hydrolase activity belong mainly to Group III. There are two conserved domains in XTH proteins, named Glyco_hydro_16 and XET_C. The XET_C domain distinguishes the XTH proteins from other proteins in the GH16 family [8–10].

XTHs are widespread in mosses, lycophytes, ferns, angiosperms and gymnosperms [9, 11–14].. Recently, XTH has even been found in algae [12]. XTHs have been widely reported in many plants, including *Arabidopsis thaliana* (33 genes), *Oryza sativa* (29 genes), *Populus* spp. (41 genes), *Solanum lycopersicum* (25 genes), *Nicotiana tabacum* (56 genes), *Glycine max* (61 genes), *Hordeum vulgare* (24 genes) and *Ananas comosus* (24 genes) [13–20]. *XTH* genes show a diversity of tissue expression. In Arabidopsis, *AtXTH1*, *AtXTH21*, *AtXTH22*, *AtXTH30* and *AtXTH33* are expressed mainly in green siliques, *AtXTH24* and *AtXTH32* mainly in stems [13]; *AtXTH9* is preferentially expressed in flower buds and flower branches, and mutation of this gene resulted in short internodes [21]. XTH proteins are active in the elongation regions of roots and hair cells of vascular plants [22]. Seven *XTH* genes in rice were found to be specifically expressed in seedling roots [14]. *DcXTH2* and *DcXTH3* from *Dianthus caryophyllus* are expressed mainly in petals [23]. XTH activity was detected during fruit expansion in *Solanum lycopersicum*, *Malus domestica*, *Actinidia chinensis,* and strawberry (*Fragaria* × *ananassa Duch*) [24–26]. Constitutive expression of *Brassica campestris BcXTH1* caused elongation of flowering branches and increased height in transgenic Arabidopsis plants [27]. Overexpression of cotton (*Gossypium spp*) *GhXTH1* improves cotton fiber length compared with that of wild type plants [28]. XTHs in *Ananas comosus* are involved in the regulation of fruit ripening and crassulacean acid metabolism and they show tissue specificity [20]. These studies all indicated that XTH is closely linked to plant growth and development.

*XTH* genes are also associated with plant stress resistance. Overexpression of the *Capsicum annuum XTH* gene *CaXTH3* enhanced drought and salt tolerance, accompanied by an increase in the number of mesophyll cells and changes in leaf shape, in transgenic Arabidopsis and pepper plants [29, 30]. Overexpression of *PeXTH* from *Populus euphratica* in tobacco plants increased their capacity for salt and Cd tolerance [31, 32]. Overexpression of *XTH* from rose (*Rosa rugosa*) enhanced drought resistance in transgenic plants [33]. The T-DNA insertion mutants *xth31*, *xth17* and *xth15* were more aluminum resistant than the wild type in Arabidopsis [34]. *MtXTH3* was induced by Hg exposure in *Medicago truncatula* [26]. Some *XTH* genes are also regulated by hormones, such as gibberellin, brassinosteroids, ethylene and auxin [26].

The *Brassicaceae* are a large family of plants and many *Brassica* species are used as oilseed crops, vegetables or feed crops around the world. The *Brassica* ancestor diverged from a common ancestor with *A. thaliana* approximately 20 million years ago (Mya) followed by a whole genome triplication (WGT) approximately 15.9 Mya. Then the *Brassica* ancestor diverged to form the modern *B. rapa* and *B. oleracea* about 3.75 Mya [35–38]. The WGT event brought an increase in genomic materials in *Brassica* species, making them an excellent model with which to investigate the expansion and evolution of gene families. In addition to genome duplication, tandem duplication (TD) is another important mechanism that induces an increase in the number of members of gene families, i.e. causes gene family expansion [39].

*B. rapa* and *B. oleracea* are important diploid species in the genus *Brassica* with completed genome sequencing projects and publicly released genome data, as well as being important as vegetables around the world [40, 41]. Although Behar et al. identified 48 and 27 XTHs in *B. rapa* and *B. oleracea* respectively by mining the JGI Phytozome v12.1 database [9], their characteristics are still unclear. In the present study we identified more XTHs in the genomes of *B. oleracea* and *B. rapa* using data from different genome versions/resources in three ways. The phylogenetic relationships, gene structures, chromosome locations, subgenome distributions, and protein sequences and tissue expression patterns of the XTHs were then analyzed, laying a foundation for further study of *XHT* gene function in *Brassica* species and providing useful information for gaining a better understanding of the function and evolution of this gene family in higher plants; the findings may also help researchers to select the most appropriate targets for further genetic engineering and genetic improvement of *Brassica* crops.

## Results

### Identification and characterization of *XTH*s

Compared with the 48 and 27 XTHs in *B. rapa* and *B. oleracea* that have previously been reported [9], we identified 53 and 38 XTHs, which include some novel members of the family, while Bol012212 was filtered out in this study because of a lack of the XET_C domain. These genes were designated corresponding to the orthologous *XTH* genes in Arabidopsis (*AtXTH*) (Table 1). The identity of BraXTHs and their Arabidopsis orthologs ranged from 61 to 96%, while, the identity of BolXTHs and their Arabidopsis orthologs varying between 57 and 95% (Additional file 1). Where the final lowercase letter in the gene name is “a”, this indicates the highest homology with Arabidopsis, “b” indicates the next highest homology, and so on. The capital letter A or C in the name indicates, respectively, the *B. rapa* Ar genome or the *B. oleracea* Co genome. The comparison results of BraXTHs reported in this paper and BraXTHs by Behar et al. [9] are shown in Additional file 2.

**Table 1:** Characteristics of XTHs identified in *B. rapa* and *B. oleracea*

Symbol	BRAD ID	Peptide length (aa)	PI	MW (kDa)	SignalP	Subcellular localization
*BraA.XTH2.a*	Bra001434	288	9.1	32.68	S	Cell wall
*BraA.XTH2.b*	Bra001433	284	8.5	31.94	S	Cell wall
*BraA.XTH3*	Bra015093	473	8.72	55.10	S	Cell wall Endoplasmic reticulum
*BraA.XTH4*	Bra013164	295	8.91	34.06	S	Cell wall Cytoplasm.
*BraA.XTH5.a*	Bra006220	293	9.21	34.09	S	Cell wall Cytoplasm.
*BraA.XTH5.b*	Bra008796	279	8.86	32.48	_	Cell wall Cytoplasm.
*BraA.XTH6*	Bra024415	285	6.06	32.75	S	Cell wall
*BraA.XTH7*	Bra017855	292	7.59	33.41	S	Cell wall
*BraA.XTH8*	Bra019846	299	5.43	35.03	S	Cell wall
*BraA.XTH9.a*	Bra000840	290	5.15	33.12	S	Cell wall
*BraA.XTH9.b*	Bra034193	344	5.06	38.88	_	Cell wall
*BraA.XTH10*	Bra040716	297	8.21	34.49	S	Cell wall
*BraA.XTH11.a*	Bra019552	301	8.66	35.11	S	Cell wall
*BraA.XTH11.b*	Bra029907	278	8.7	32.59	S	Cell wall
*BraA.XTH12.a*	Bra002723	286	5.4	32.42	S	Cell wall Cytoplasm.
*BraA.XTH12.b*	Bra020436	283	5.11	32.24	S	Cell wall Cytoplasm.
*BraA.XTH12.c*	Bra006814	319	5.43	36.29	S	Cell wall Cytoplasm.
*BraA.XTH14.a*	Bra019141	286	6.95	32.69	S	Cell wall Cytoplasm.
*BraA.XTH14.b*	Bra013923	284	5.96	32.20	S	Cell wall Cytoplasm.
*BraA.XTH15*	Bra038442	289	9.41	33.03	S	Cell wall Cytoplasm.
*BraA.XTH16*	Bra014975	291	9.14	33.20	S	Cell wall Cytoplasm.
*BraA.XTH17.a*	Bra011181	282	8.56	32.01	S	Cell wall
*BraA.XTH17.b*	Bra011180	282	9	32.10	S	Cell wall
*BraA.XTH17.c*	Bra010290	280	9.3	32.03	S	Cell wall
*BraA.XTH17.d*	Bra024087	284	8.84	32.36	S	Cell wall
*BraA.XTH17.e*	Bra010291	282	9.1	32.23	S	Cell wall
*BraA.XTH18*	Bra024088	282	9.09	32.25	S	Cell wall
*BraA.XTH21*	Bra038819	239	9.12	27.27	_	Cell wall
*BraA.XTH22.a*	Bra002719	283	5.96	31.98	S	Cell wall Cytoplasm.
*BraA.XTH22.b*	Bra020433	260	5.49	29.61	S	Cell wall
*BraA.XTH22.c*	Bra002718	214	6.72	24.39	_	Cell wall Cytoplasm.
*BraA.XTH22.d*	Bra002720	214	6.72	24.39	_	Cell wall Cytoplasm.
*BraA.XTH22.e*	Bra020432	283	6.33	31.79	S	Cell wall
*BraA.XTH23.a*	Bra008806	287	5.29	32.11	S	Cell wall Cytoplasm.
*BraA.XTH23.b*	Bra013922	287	5.29	32.11	S	Cell wall Cytoplasm.
*BraA.XTH24.a*	Bra011179	282	8.76	31.84	S	Cell wall Cytoplasm.
*BraA.XTH24.b*	Bra024089	279	8.93	31.59	S	Cell wall Cytoplasm.
*BraA.XTH24.c*	Bra010292	212	8.76	24.37	_	Cell wall Cytoplasm.
*BraA.XTH25.a*	Bra002722	284	9.06	32.58	S	Cell wall Cytoplasm.
*BraA.XTH25.b*	Bra020434	285	7.66	32.49	S	Cell wall Cytoplasm.
*BraA.XTH26*	Bra011066	292	9.51	33.07	S	Cell wall
*BraA.XTH27.a*	Bra026630	333	7.67	38.29	S	Cell wall
*BraA.XTH27.b*	Bra024848	323	7.73	36.95	_	Cell wall
*BraA.XTH28*	Bra026809	328	7.24	37.79	S	Cell wall
*BraA.XTH29.a*	Bra012559	347	9.1	40.07	S	Cell wall
*BraA.XTH29.b*	Bra013367	351	8.68	40.49	S	Cell wall
*BraA.XTH30*	Bra035513	342	8.14	39.74	S	Cell wall
*BraA.XTH31.a*	Bra019416	292	7.04	33.11	S	Cell wall
*BraA.XTH31.b*	Bra037614	292	8.21	33.57	S	Cell wall
*BraA.XTH32.a*	Bra005238	289	9.58	34.30	S	Cell wall
*BraA.XTH32.b*	Bra017220	299	9.53	34.15	S	Cell wall
*BraA.XTH32.c*	Bra023083	299	9.49	34.25	S	Cell wall
*BraA.XTH33*	Bra018433	317	8.97	35.38	S	Cell wall
*BolC.XTH2*	**Bol003021**	291	8.69	32.92	S	Cell wall
*BolC.XTH4*	Bol042558	295	8.91	34.12	S	Cell wall Cytoplasm.
*BolC.XTH5*	Bol043401	295	8.87	34.27	S	Cell wall Cytoplasm.
*BolC.XTH7*	Bol018592	292	7.6	33.40	S	Cell wall
*BolC.XTH8*	**Bol036640**	199	4.96	23.32	S	Cell wall
*BolC.XTH9.a*	Bol030618	290	5.15	33.09	S	Cell wall
*BolC.XTH9.b*	Bol006115	290	5.02	33.04	S	Cell wall
*BolC.XTH11.a*	**Bol013144**	209	9.32	24.68	–	Cell wall
*BolC.XTH11.b*	**Bol013146**	225	8.21	26.08	S	Cell wall
*BolC.XTH12*	Bol026029	286	5.37	32.43	S	Cell wall Cytoplasm.
*BolC.XTH13*	Bol012213	268	5.72	30.74	_	Cell wall Cytoplasm.
*BolC.XTH15*	Bol027057	289	9.34	33.01	S	Cell wall Cytoplasm.
*BolC.XTH16*	Bol037310	291	9.14	33.18	S	Cell wall Cytoplasm.
*BolC.XTH17.a*	Bol020988	282	9.01	32.12	S	Cell wall
*BolC.XTH17.b*	Bol033655	248	8.74	28.50	S	Cell wall
*BolC.XTH20*	**Bol012994**	187	9.56	21.28	S	Cell wall
*BolC.XTH21*	Bol041548	340	8.91	38.61	_	Cell wall
*BolC.XTH22.a*	Bol014220	283	5.8	32.07	S	Cell wall Cytoplasm.
*BolC.XTH22.b*	Bol014219	283	5.98	31.76	S	Cell wall
*BolC.XTH23*	Bol039563	287	5.05	32.12	S	Cell wall Cytoplasm.
*BolC.XTH24.a*	Bol033653	279	8.77	31.59	S	Cell wall Cytoplasm.
*BolC.XTH24.b*	Bol012996	323	8.95	36.96	S	Cell wall Cytoplasm.
*BolC.XTH24.c*	**Bol033652**	284	5.07	32.35	S	Cell wall
*BolC.XTH24.d*	Bol020987	212	8.6	24.38	_	Cell wall Cytoplasm.
*BolC.XTH25*	Bol014221	285	7.67	32.39	S	Cell wall Cytoplasm.
*BolC.XTH26*	Bol019625	292	9.5	33.08	S	Cell wall
*BolC.XTH27.a*	Bol004698	346	8.2	39.87	S	Cell wall
*BolC.XTH27.b*	**Bol002411**	269	9.12	30.57	S	Cell wall
*BolC.XTH28*	Bol031516	318	7.22	36.36	S	Cell wall
*BolC.XTH29.a*	**Bol009357**	271	8.64	31.02	S	Cell wall
*BolC.XTH29.b*	**Bol024395**	163	9.3	18.67	_	Cell wall
*BolC.XTH30.a*	**Bol001946**	343	6.55	39.68	S	Cell wall
*BolC.XTH30.b*	Bol014054	342	7.62	39.70	S	Cell wall
*BolC.XTH31*	Bol041311	292	7.65	33.21	S	Cell wall
*BolC.XTH32.a*	Bol037699	299	9.57	34.11	S	Cell wall
*BolC.XTH32.b*	**Bol039723**	243	9.75	27.84	S	Cell wall
*BolC.XTH32.c*	**Bol001671**	234	9.82	26.78	S	Cell wall
*BolC.XTH33*	Bol022041	317	8.82	35.50	S	Cell wall

New homologs identified in this study in *B. oleracea* are shown in bold. The rest BolXTHs have been identified by Behar et al. [9]

No orthologs of *AtXTH1*, *AtXTH2*, *AtXTH6*, *AtXTH10*, *AtXTH14*, *AtXTH18* or *AtXTH19* were found in the *B. oleracea* genome, while the genome of *B. rapa* lacked orthologs of *AtXTH1*, *AtXTH3*, *AtXTH19* and *AtXTH20.* Thus more *XTH* genes have been lost from *B. oleracea* than from *B. rapa*.

The lengths of BraXTHs ranged from 212 (BraA.XTH24.c) to 473 (BraA.XTH3) amino acids, with the molecular weights varying between 24.37 kDa to 55.10 kDa, while, the length of BolXTHs ranged from 163 (BolC.XTH29.b) to 346 (BolC.XTH27.a) amino acids, with the molecular weights varying between 18.67 kDa and 39.87 kDa. BraXTH3 was the largest XTH protein in this study. It possesses an ER lumen protein retaining receptor (ER_lumen_recept: InterPro IPR000133, Pfam PF00810) domain in the N-terminal compared with other identified XTHs.

The theoretical PI values for XTHs ranged from 5.06 to 9.58 in *B. rapa* and 4.96–9.75 in *B. oleracea* due to the differences in the polarities of the amino acids making up these proteins. The numbers of introns in *XTH* genes were relatively similar in the two species; 86.8% of *BraXTH* genes and 89.5% of *BolXTH* genes had 2–3 introns, of which 24 *BraXTHs* and 19 *BolXTHs* had 3 introns, and 22 *BraXTHs* and 15 *BolXTHs* had 2 introns. The number of introns in *BraA.XTH3* was the largest (7), while *BolC.XTH29.b* lacked introns.

The Plant-mPLoc server (http://www.csbio.sjtu.edu.cn/bioinf/plant-multi/) was used to predict the subcellular location of BraXTH and BolXTH proteins. The result showed that all XTH proteins were located on the cell wall. In addition to the cell wall, 20 BraXTHs and 12 BolXTHs were also predicted to localize in the cytoplasm. BraA.XTH3 was found to be located in both the cell wall and the endoplasmic reticulum. XTH localize just were bioinformatic speculation and the real situation will be experimental evidence. The signal peptide prediction results indicated that 46 BraXTHs and 33 BolXTHs had signal peptides.

### Phylogenetic analysis of XTH proteins

**Fig. 1:**
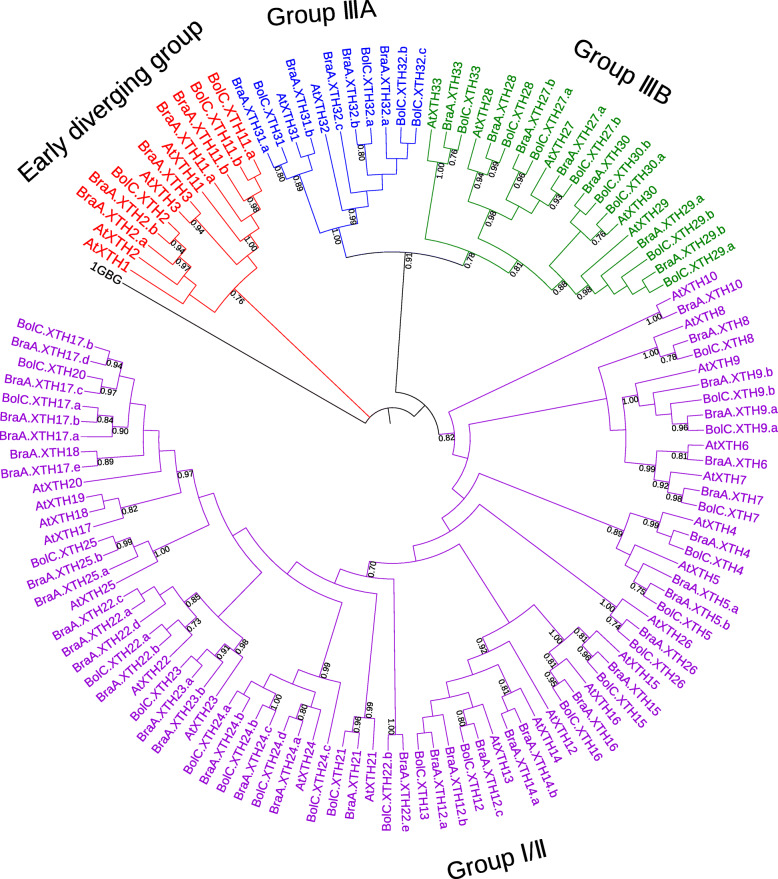
Phylogenetic analysis of full-length XTH proteins in *B. rapa*, *B. oleracea* and Arabidopsis. The tree was constructed by the Maximum Likelihood (ML) method using MEGA7, with the JTT + G model and 1000 bootstrap replicates. The branches correspond to the three phylogenetic groups

In order to investigate the evolutionary relationship among different *XTH* gene family members, we used the full-length XTH protein sequences from *B. rapa*, *B. oleracea* and *A. thaliana* to generate a phylogenetic tree based on the Maximum Likelihood method, using a structurally characterized bacterial lichenase (1GBG, EC 3.2.1.73) as an outgroup (Fig. 1, Additional file 3). Three groups (Early diverging group, Group I/II and Group III) were identified based on clade support values, the topology of the phylogenetic tree, and the previous classification of XTH families in Arabidopsis [6, 13]. So far, XEH activity has only been reported in clade IIIA [9, 42]. The early diverging close to the root was the smallest group**,** containing 12 members. There were 11 XTHs in Group IIIA and 20 in Group IIIB. The rest of the XTHs belonged to Group I/II, which included 22 AtXTHs, 35 BraXTHs and 23 BolXTHs. As Fig. 1 shows, XTHs from *B. rapa* and *B. oleracea* were clustered with their *A. thaliana* homologs. There were 41 sister pairs at the termini of phylogenetic tree branches that showed close relationships and 30 of these were orthologous pairs between the *B. rapa* genome and the *B. oleracea* genome.

### Structure of *XTH* genes, pattern of motifs and structure-based sequence alignment in XTH proteins

**Fig. 2:**
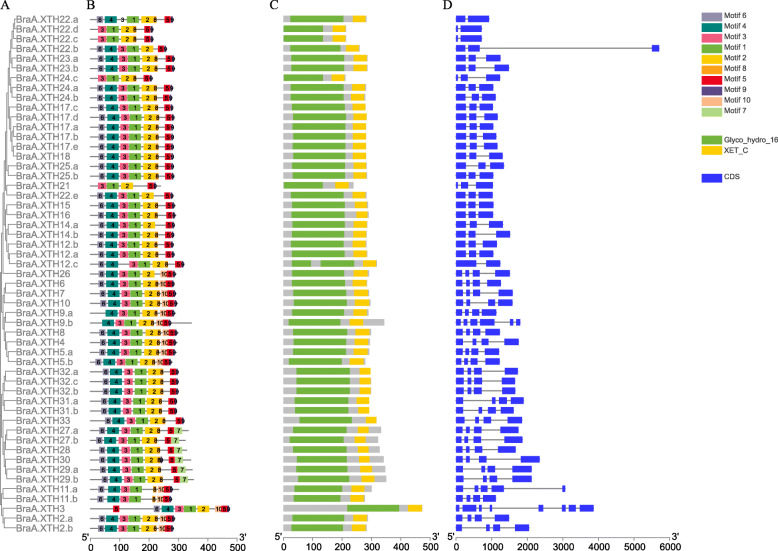
Characterizations of *XTH* gene family members in *B. rapa*, including phylogenetic tree (**a**), conserved motif location (**b**), domain location (**c**) and intron/exon structure (**d**)

**Fig. 3:**
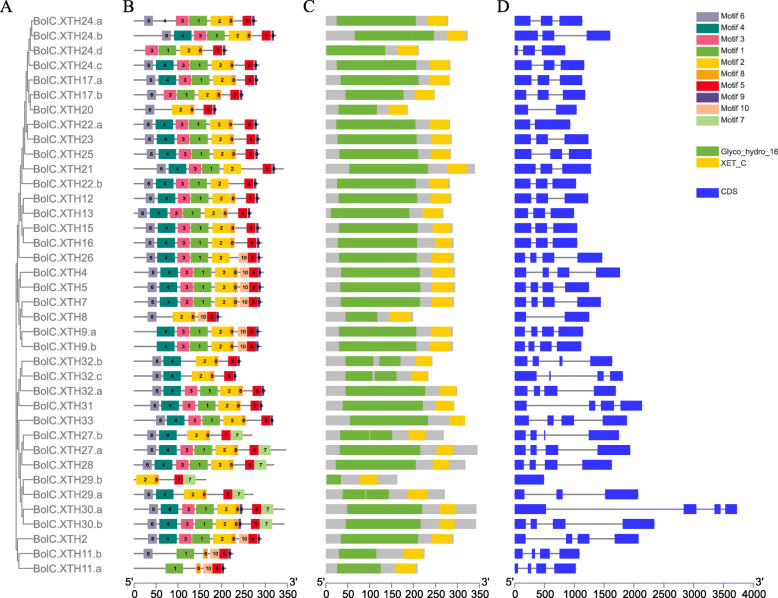
Characterizations of *XTH* gene family members in *B. olerace,* including phylogenetic tree (**a**), conserved motif location (**b**), domain location (**c**) and intron/exon structure (**d**)

Previous studies showed that the exon organization in Arabidopsis XTH genes is well conserved within each group [13, 43]. To better characterize the structural conservation and diversification of *XTH* genes during their evolution, the exon-intron organization of the coding sequences of individual *XTH* genes coding sequence was obtained for members of each group. Each XTH protein in the two species had a Glyco_hydro_16 domain and an XET_C domain. As shown in Fig. 2b, c and Fig. 3b, c, the Glyco_hydro_16 domain spanned the sequence of motifs 6–4–3-1-2-8, though some proteins lacked one or more of these motifs. The lengths of 4 BraXTHs and 9 BolXTHs, including 7 newly identified XTHs, are less than 250 amino acids, due to the deletion of 1 to 4 motifs from the Glyco_hydro_16 domain (Figs. 2, 3). The XET_ C domain mainly covered motifs 5 and 9. Fifteen BraXTHs and 10 BolXTHs also shared motif 10, forming the block 10–5-9. Six BraXTHs and 7 BolXTHs replaced motif 9 with motif 7, forming a new tandem motif pattern (motif 5–7 in tandem). Overall, motifs had a similar distribution within the same group.

In addition to *XTH26*, all genes in Group I contained 1–2 introns. Apart from *XTH8*, all genes in Group II contained 3 introns. All Group III genes in the two species had 3 introns with the exceptions of *BolC.XTH29.a* and *BolC.XTH29.b*. Generally, the motif patterns in different XTH proteins showed only small differences, and the genes that clustered in the same group showed similar patterns of gene structure.

The alignments of the XTHs together with PttXET16A (PDB id: 1UN1), a xyloglucan endotransglycosylase with known protein structure [44, 45], were used to predict the secondary structures of the BraXTH proteins and BolXTH proteins using ESPript (http://espript.ibcp.fr/ESPript/ESPript/) (Additional files 4 and 5). The position of the N-glycosylation site of PttXET16A and BobXET16A with known protein structure, was conserved [44–46]. The site also was conserved in 46 BraXTHs and 28 BolXTHs, but it was not found in 7 BraXTHs and 10 BolXTHs: BolC.XTH31, BolC.XTH32.a, BolC.XTH33, the 7 BolXTHs that lacked the EXDXE conserved active-site motif (BolC.XTH8, BolC.XTH20, BolC.XTH27.b, BolC.XTH29a\b and BolC.XTH32.b\c), BraA.XTH2.a, BraA.XTH31.a\b, BraA.XTH32.a\b\c and BraA.XTH33 (Additional files 4 and 5). Alterations of amino acid residues were found within this catalytic region in AtXTH11 and its homologs. In AtXTH11, EXDXE was replaced by ELCFQ, while it was replaced by GLCFQ in BraA.XTH11b and BolC.XTH11.a\b, and by QLCFQ in BraA.XTH11.a. Though XTH proteins identified in this study contained two characteristic conserved domains (Glyco_hydro and XET_C) by searching Pfam database, some XTHs lacked one or several α-helices or/and β-strands compared with PttXET16A. Comparative analysis showed motif 6 covered α1-helices, β1-β2 strands, motif 4 covered β4, part of β3 and β5, motif 3 covered β6 and part of β5, motif 1 covered β7–8, motif 2 covered β9–12, motif 8 covered β 13–14, motif 5 covered α1 and β 15, respectively. There is no uniform correspondence between motif and α-helices or/and β-strands.

### Chromosomal distribution and duplication analysis of *XTH* genes

**Fig. 4:**
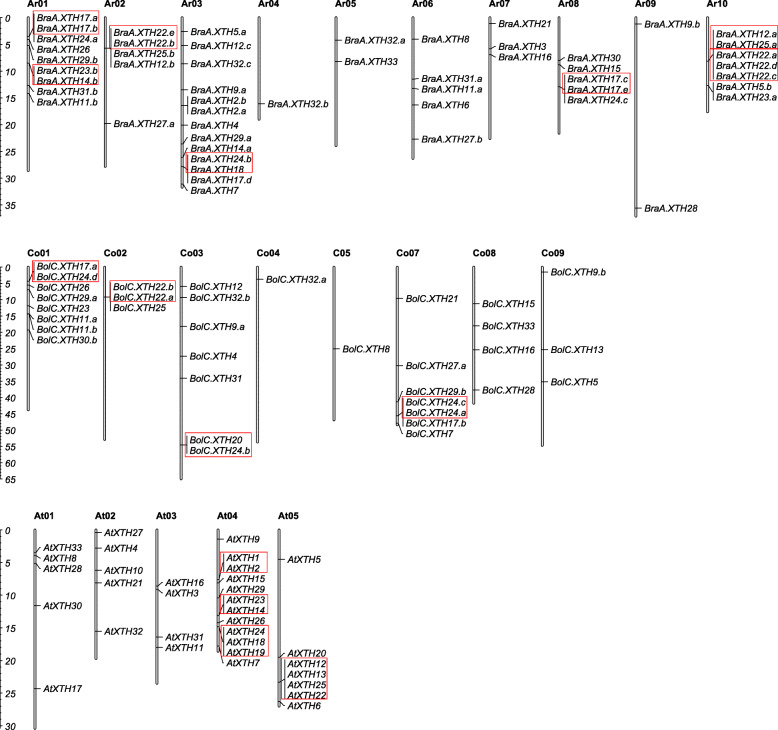
Distribution of *XTH* genes on *B. rapa*, *B. oleracea* and *A. thaliana* chromosomes. *XTH* genes in red boxes are tandemly repeated genes. The number of chromosomes was indicated at the top of each chromosome. Ar, Co and At in front of number represent chromosome in *B. rapa*, *B. oleracea* and *A. thaliana*, respectively. The scale on the left is in megabases (Mb)

The chromosomal locations of all *XTH* genes in both *Brassica* species were investigated based on their physical positions and are shown in Fig. 4. Excluding *BraA.XTH10*, which was positioned on a scaffold*,* the remaining fifty-two *BraXTH* genes had definite chromosomal locations; mapping onto the different chromosomes was uneven. Chromosome Ar03 in *B. rapa* carried the greatest number of genes (13), while Ar04 carried only one *XTH* gene. In *B. oleracea,* there were 34 *XTH* genes with definite locations and they were distributed among all chromosomes excluding chromosome Co06. Chromosome Co01 was a “hot region”, carrying the greatest number of genes (8); in contrast Co04 and Co05 each contained only one *XTH* gene. Incomplete genome assembly meant that definite chromosomal locations were not available for five XTHs: *BraA.XTH10*, *BolC.XTH2*, *BolC.XTH27.b*, *BolC.XTH30.a* and *BolC.XTH32.c*.

TD events contribute to the expansion of gene families and can produce tandemly repeated genes in clusters [47]. We obtained putative tandemly-duplicated *XTH* genes of the two *Brassica* species from PTGBase. As a result, 15 *BraXTH* genes and 8 *BolXTH* genes were found to be present in tandem arrays, representing 28.3 and 21.1% of the total *XTH* genes in *B. rapa* and *B. oleracea* respectively. These tandemly repeated genes were clustered, which was consistent with their chromosomal locations (Fig. 4). Seven tandem arrays were identified on chromosomes Ar01, Ar02, Ar03, Ar08 and Ar010 in *B. rapa*. Protein BLAST analysis revealed that BraA.XTH17.a is 93% identical to BraA.XTH17.b, BraA.XTH22C is 99% identical to BraA.XTH22.c or BraA.XTH22.d, and BraA.XTH22.a is 100% identical and 75% coverage to BraA.XTH22.d. The identity of the other tandem gene pairs is varying from 55 to 68%. Four tandem arrays occurred on Co01, Co02, Co03 and Co07 in *B. oleracea*, with 58 to 84% identity of tandem gene pairs.

In *A. thaliana*, four tandemly duplicated gene arrays composed of nine *AtXTH*s were found (Fig. 4). Tandem arrays including *AtXTH1*/*2*, *AtXTH23*/*14* and *AtXTH24*/*18*/*19* were located on chromosome At04 while *AtXTH12*/*13*/*25*/*22* was on chromosome At05. It is worth mentioning that some genes that bear syntenic relationships to these tandem genes, though not *AtXTH1*/*2*, have a conserved tandem repeat pattern in both the *B. rapa* genome and the *B. oleracea* genome, suggesting that these tandem arrays arose before the divergence of *A. thaliana* and the *Brassica* ancestor.

### Syntenic analyses of *XTH* genes

The ancestor of diploid *Brassica* species experienced a WGT event since divergence from the Arabidopsis lineage. Syntenic genes are orthologous genes located in fragments syntenic between different species that derive from a shared ancestor, and synteny analysis can be used to transfer gene annotations and investigate genomic evolution in related species [48]. We obtained the genes syntenic with the *XTH* genes of Arabidopsis for the two *Brassica* species by searching for ‘syntenic gene’ in BRAD (Additional file 6). According to comparative genomics analysis, the density and expression level of genes in different regions show some differences in the genomes of *B. rapa* and *B. oleracea*, which can be divided into three fractionated subgenomes which we denoted LF (Least-fractionated), MF1 (Medium-fractionated), and MF2 (Most-fractionated) according to the extent of gene retention [41, 49]. Statistical analysis indicated that there were 13, 13, and 6 *BraXTH* genes and 9, 10, and 5 *BolXTH* genes located in the LF, MF1 and MF2 subgenomes respectively (Additional file 6). In summary, 60.4 and 63.2% of the total *XTH* genes in, respectively, *B. rapa* and *B. oleracea* were located in syntenic blocks. WGD events are therefore likely to have played a major role in the expansion of *XTH* genes in the two *Brassica* species. The identities of 75% (24 out of 32) BraXTHs and 62.5% (15 out of 24) BolXTHs with their Arabidopsis syntenic orthologs exceeded 80% (Additional file 6).

A total of 23 *AtXTH* genes had corresponding syntenic genes in the two *Brassica* species. The copy numbers of syntenic genes in the genomes of the two *Brassica* species differed. The first situation was one in which genes syntenic with *AtXTH* genes were completely preserved in the same syntenic block in the Ar and Co subgenome; 8 genes were of this type. In the second case, *AtXTH* genes were retained in the Ar genome but lost from the Co genome, this applied to *AtXTH3* and *AtXTH5*. The third case was where *AtXTH* genes had more than one syntenic gene in *B. rapa* or *B. oleracea*. For example, 8 and 1 *AtXTH* genes had 3 syntenic genes in *B. rapa* and *B. oleracea* respectively. An *AtXTH* should theoretically correspond to 3 syntenic genes and if there are fewer than 3 it may be the result of gene loss after genome replication.

### Selection forces acting on XTH duplicated pairs

To assess whether XTH duplicated pairs in *Brassica* species experienced different selective forces, Ka/Ks values were calculated (Additional file 7). A Ka/Ks ratio > 1 represents positive selection, Ka/Ks = 1 represents neutral selection and a Ka/Ks ratio < 1 represents purifying selection [50]. We found 33 and 18 segmentally duplicated XTH gene pairs in *B. rapa* and *B. oleracea* respectively. All segmentally duplicated XTH gene pairs had Ka/Ks < 1, while two tandemly duplicated gene pairs (*BraA.XTH22.a*-*BraA.XTH22.d* and *BraA.XTH22.c*-*BraA.XTH22.d*) had no Ka/Ks value in *B. rapa* because they shared the same sequence.

The segmental duplications of the *XTH* genes in *B. rapa* originated between 0.34 Mya (Ks = 0.0103) and 28.80 Mya (Ks = 0.8640), with a mean of 12.88 Mya (Ks = 0.1436). After comparative analysis, the segmental duplications of the *BolXTH* genes were found to have originated from 5.37 Mya (Ks = 0.1612) to 32.12 Mya (Ks = 0.9637), with a mean of 13.20 Mya (Ks = 0.3960). Overall, the Ka/Ks ratios for segmental duplication of *BolC.XTH11.b* and *BolC.XTH11.a*, *BraA.XTH2.b* and *BraA.XTH2.a*, together with *BraA.XTH23.a* and *BraA.XTH23.b*, were > 0.3, while the ratios for the other segmental duplication pairs were all < 0.3, suggesting that significant functional divergence of some *XTH* genes might have occurred after the duplication events.

### Expression patterns of *XTH* genes in different tissues of *B. rapa* and *B. oleracea*

To understand the variations in expression pattern for *XTH* genes, we analyzed *XTH* gene expression patterns across different tissues in the two species of *Brassica* based on RNA-Seq retrieved from the GEO database (Additional file 8). If the FPKM of a gene was less than 1, it was considered to be an unexpressed gene in this study, including *BraA.XTH2.a*/*b*, *BraA.XTH5.b*, *BraA.XTH11.a*, *BraA.XTH12.a*/*b*/*c*, *BraA.XTH25.a*/*b*, *BolC.XTH5*, *BolC.XTH11.a*, *BolC.XTH20*, *BolC.XTH21*, *BolC.XTH22.b*, *BolC.XTH24.c*, *BolC.XTH25* and *BolC.XTH26*. In addition, *BolC.XTH12* and *BolC.XTH13* lacked FPKM values. On this basis, 44 *BraXTH* genes and 28 *BolXTH* genes were expressed in at least one tissue, while the remaining genes lacked expression data or were unexpressed in all the tissues tested, indicating that they might be non-functional or have specific temporal and spatial expression patterns that were not detected in this study. There were 23 out of 53 (approximately 43.4%) *BraXTH* genes and 14 out of 38 (approximately 36.8%) *BolXTH* genes that were widely expressed in all the tissues tested (root, stem, leaf, flower, silique and callus of *B. rapa*; root, stem, leaf, flower, silique, callus and bud of *B. oleracea*). The remaining 21 *BraXTH* genes and 14 *BolXTH* genes were expressed in at least one but not in all tested tissues. For example, *BraA.XTH29.a* and *BraA.XTH29.b* were expressed specifically in the flower; *BraA.XTH10*, *BraA.XTH17.c*, *BraA.XTH17.d* and *BraA.XTH32.b* were expressed in all tissues except callus. *BolC.XTH2* was expressed solely in the silique and *BolC.XTH29.b* was expressed only in buds at low levels.

**Fig. 5:**
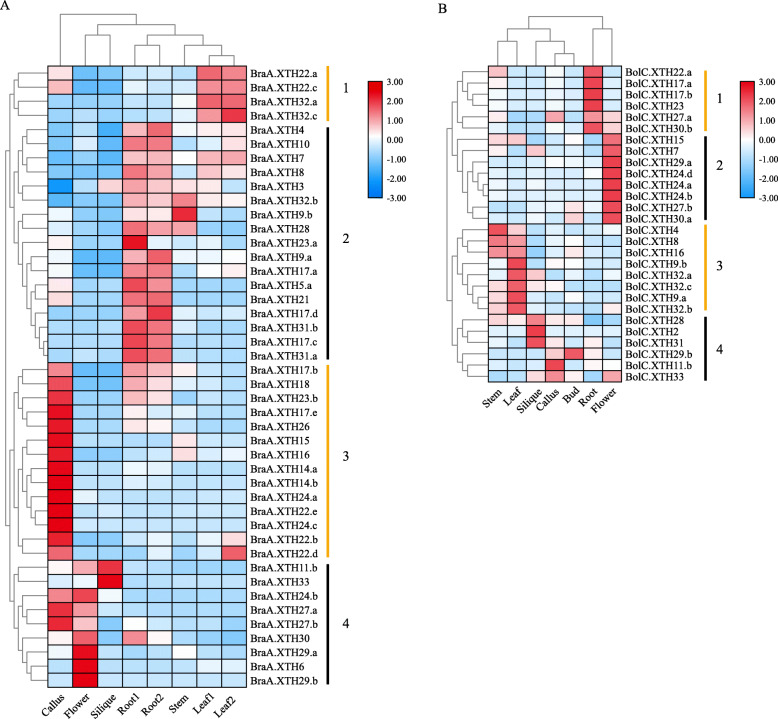
Heatmaps of expression clustering for *XTH* genes across different tissues in *B. rapa* (**a**) and *B. oleracea* (**b**) based on FPKM values retrieved from the GEO database (http://www.ncbi.nlm.nih.gov/geo/). Two samples of root and leaf tissues were generated from different batches of plants for Fig. 5a. Color scale bars representing relative signal ratios are shown at the top of the right-hand side of each heatmap. Heatmaps were drawn with TBtools. The relative level of expression of a particular gene in each row was normalized against the mean value. Euclidean distance was used to evaluate closeness between genes, and the average linkage method was used for cluster analysis

Clustering analysis of expression values showed that both the *B. rapa* and the *B. oleracea XTH* genes can be divided into four groups (Fig. 5). In *B. rapa*, *XTH* genes in cluster 1 were more highly expressed in the leaf than in the other tissues examined, while cluster 2 were expressed mainly in the root, apart from *BraA.XTH32.b* and *BraA.XTH9.b*. Cluster 3 showed higher expression in callus and group 4 was expressed mainly in flower, silique or callus. In *B. oleracea*, *XTH* genes in cluster 1 were highly expressed in the root, whereas cluster 2 was expressed mainly in the flower. Four genes in cluster 3 were expressed mainly in the stem or leaf and genes in cluster 4 were expressed mainly in the leaf, silique or callus. *XTH* genes in the same group based on phylogenetic analysis did not show the same expression patterns.

Some tandemly repeated family members, such as *BraA.XTH22.a* and *BraA.XTH22.c* in cluster 1, showed similar expression patterns across the tissues tested, indicating the possible existence of redundancy (Fig. 5a). However, most tandemly repeated members displayed distinct expression patterns. For example, *BolC.XTH24.a* and *BolC.XTH24.b* showed higher expression levels in the flower than the other tissues, whereas tandem repeats of them, B*olC.XTH24.c* and *BolC.XTH20*, were not expressed in these tissues. *BolC.XTH17.a* showed high expression in the root and low expression in the bud, leaf and silique, while *BolC.XTH24.d* showed high expression in the flower and low expression in the leaf (Fig. 5, Additional file 8). All *XTH* tandem genes in seven arrays were also analyzed and compared in *B. rapa*. A total of 2 tandem genes (*BraA.22b*/*e* and *BraA.14b*/*23b*) showed different abundances, but the same trend with respect to patterns, whereas the two members of each of the other pairs of tandem genes showed differences in abundance and tissue specificity of expression. In general, *XTH* genes in the two *Brassica* species exhibit differential patterns of expression across different tissues, leading to different functional clusters and suggesting functional divergence.

## Discussion

### The *XTH* gene family expanded in *B. rapa* and *B. oleracea*

Previous studies revealed that the *Brassica* genome, like that of *A. thaliana*, underwent three paleo-polyploidy events. *Brassica* species also shared an additional WGT event since isolation from Arabidopsis [40, 41]. Compared to the 33 *AtXTH* genes [13], higher numbers of *XTH* genes were identified in the *B. rapa* (53 genes) and *B. oleracea* (38) genomes. Moreover, 60.4 and 63.2% of the total *XTH* genes in, respectively, *B. rapa* and *B. oleracea* were located in syntenic blocks. WGD events therefore played a major role in the expansion of *XTH* genes in these two *Brassica* species. The secondary force leading to the expansion of *XTH* genes was tandem duplication. There were, respectively, 28.3 and 21.1% of the total *XTH* genes that were involved in tandem arrays in *B. rapa* and *B. oleracea*, and *B. rapa* has more tandem *XTH* genes than *B. oleracea,* which indicated that *B. oleracea* has lost some of its XTH tandem genes during the process of gene duplication. It reflects the amplification of tandem repeat genes is asymmetric between the two species. Tandem duplication also contributed to *XTH* gene family expansion in barley, tobacco, sorghum, and soybean [15, 19, 51, 52]**.**

Since the *Brassica* ancestor diverged from its common ancestor with *A. thaliana* ~ 20 Mya; it subsequently underwent a WGT event ~ 15.9 Mya. Then the *Brassica* ancestor diverged to form the modern *B. rapa* and *B. oleracea* about 3.75 Mya [35–38]. In this study, we found that most of the segmental duplications of *BraXTH* and *BolXTH* genes occurred before the divergence of the modern *B. rapa* and *B. oleracea.* However, *BraA.XTH16* and *BraA.XTH15* arose around 28.80 Mya before the divergence of the *Brassica* ancestor and its common ancestor with *A. thaliana,* while *BraA.XTH23.a* and *BraA.XTH23.b* arose around 0.34 Mya after the divergence of the modern *B. rapa* and *B. oleracea.*

### The *XTH* gene family is highly conserved at the DNA and protein level

The XTH proteins of *A. thaliana*、*B. rapa* and *B. oleracea* can be divided into 3 groups according to the results of phylogenetic analysis. The number of genes in Group IIIA is the smallest, while the number in Group I/II is the largest, which is consistent with results obtained from other plants [20]. All XTH proteins were found to be located at the cell walls and this positioning is consistent with the function of XTH proteins involved in cell wall reconstruction. In addition, some XTHs, all members of Group I/II, were also found to be located in the cytoplasm. These findings were similar to those reported in barley and pineapple [15, 20]. Interestingly, the TD genes all belong to Group I/II, suggesting that TD is the reason for the large number of genes in this group. It has been reported that proteins showing XET activity belong mostly to Groups I/II and IIIB, while proteins showing XEH activity belong mostly to group IIIA [3, 9, 26]. Thus *Brassica* XTH members in different groups may show different types of enzyme activity. Unlike the proteins in other groups, the Group IIIB proteins of the two species contained motif 7, suggesting that this motif may be related to the specific function(s) of the IIIB proteins.

### Gene and domain loss events in the XTH family

The genome size of *B. rapa* and *B. oleracea* is different, at about 529 Mb and 696 Mb respectively, and the total number of genes according to the PLAZA 4.5 database is about 42,000 and 45,000 respectively [27]. However, *B. rapa* apparently has more *XTH*s than *B. oleracea*; this may be because the *B. oleracea* genome assembly was incomplete, leading to incomplete identification, or may be due to a greater loss of *XTH* genes from *B. oleracea.*

Compared with *A. thaliana*, the *Brassica* genome experienced a unique triplication event [40, 41]. Hence, each XTH gene in Arabidopsis should correspond to three homologs in *B. rapa* and *B. oleracea*. However, the number of *XTH* genes obtained from each of the two species was far less than three times the number of *XTH* genes in *A. thaliana*. All the *AtXTH* genes had 0–2 orthologs in the genomes of the two species, apart from *AtXTH32*, which had three homologs. This indicates that the loss of *XTH* genes occurred after the *Brassica* WGT event. Synteny analysis revealed that 61.5% of the *XTH* genes of the two *Brassica* species were located in conserved chromosomal blocks, whereas some genes had been deleted. These syntenic blocks account for the majority of the *XTH* genes in *A. thaliana* (75.76% of the genes), *B. oleracea* (63.16%) and *B. rapa* (84.21%). At the whole genome level syntenic blocks contain 72.24, 57.88 and 64.84% of the genes in *A. thaliana*, *B. oleracea* and *B. rapa* respectively [41]. *B. oleracea* had lost a considerably greater number of *XTH* genes compared with *B. rapa*, consistent with the asymmetry of gene loss between the two genomes.

After carrying out comparative analysis of the pattern of retention/loss of orthologous genes in each set of three subgenomic (LF, MF1 and MF2) blocks of the two species corresponding to *A. thaliana,* we found the *XTH* genes retained in *B. rapa* and *B. oleracea* are mostly located in the LF subgenome and the MF1 subgenome, which is consistent with the retention pattern for their genomes as a whole. The MF2 subgenome retained the fewest *XTH* genes and has thus undergone the greatest loss of orthologous genes. The MF1 subgenome had lost the largest number of genes at the whole genome level. There were differences in the levels of XTH loss among the three subgenomic (LF, MF1 and MF2) blocks, which was consistent with the difference in gene loss rate among subgenomes [41].

Previous research demonstrated that statistically, more than one-third of all domains have a marked tendency to increase/decrease in size during protein evolution [53]. XTH proteins generally contain a characteristic motif, EXDXE, which contains amino acid residues that mediate catalytic activity. Site-directed mutation of AtXTH22 has indicated that the first glutamine residue in this motif is required for catalytic activity [54]. Compared with the AtXTH protein structures, there were seven BolXTHs lacking the EXDXE conserved active-site motif: BolC.XTH8, BolC.XTH20, BolC.XTH27.b, BolC.XTH29a\b and BolC.XTH32.b\c. In addition, several proteins encoded by syntenic *XTH* genes had a Glyco_hydro_16 domain but lacked an XET_C domain, so that they could not be identified as XTHs in this study. These phenomena reflect differences in the evolution of homologous genes between the *B. rapa* genome and the *B. oleracea* genome, and the higher level of DNA loss from the *B. oleracea* genome. Domain loss has also been observed in *Hsp70* genes of *Brassica* species [55].

### The patterns of expression of *XTH* genes

Previous research using GUS staining in Arabidopsis [56] showed that *AtXTH*s are probably expressed in all developmental stages from seed germination through to flowering. In this study, 83 and 74% of the *XTH* genes in, respectively, *B. rapa* and *B. oleracea* were expressed across all the tissues examined. Comparative analysis showed that the *AtXTH* orthologs in *B. rapa* and *B. oleracea* showed different expression characteristics, even among orthologous genes with high levels of identity of amino acid sequences. In a previous study *AtXTH21*, *AtXTH22* and *AtXTH30* were found to be expressed mainly in siliques [13]. However, their orthologs in the two *Brassica* species exhibited different expression patterns. For example, *BraA.XTH21* had only a low expression level in callus and roots, but *BolC.XTH21* was not express; *BolC.XTH22.a* was expressed mainly in roots, stems and callus, while *BolC.XTH22.b* was not expressed in any of the tissues examined; *BraA.XTH30* showed high expression levels in callus, roots and flowers, while *BolC.XTH30.a* showed its highest expression level in flowers, and *BolC.XTH30.b* was expressed most highly in roots. *BraA.XTH33* showed the highest expression level in siliques, consistent with its Arabidopsis homolog (*AtXTH33*), but *BolC.XTH33* was expressed in callus, silique and flower at an intermediate level. *BolC.XTH24.a*/*b*/*d* all showed the highest expression levels in flowers but *BolC.XTH24.c* showed no expression in this tissue, *BraA.XTH24.a/b/c* showed their highest expression levels in callus, followed by flowers, whereas the expression of its Arabidopsis homolog (*AtXTH24*) was mainly in stems [13].

Duplicated genes usually share high levels of sequence similarities; however, over the course of evolution, the fates of duplicated genes may be quite different, as they undergo nonfunctionalization, neofunctionalization or subfunctionalization [57]. As a result of comparative analysis of the expression profiles of *XTH* genes involved in tandem and segmental duplications, patterns of similar, different or silenced gene expression relative to other members were found. For example, *BraA.XTH32.a* and *BraA.XTH32.c*, *BraA.XTH14.a* and *BraA.XTH14.b*, which were segmentally duplicated genes, exhibited similar expression behavior, indicating that their roles may have been conserved after the duplication events. *BraA.XTH23.a* and *BraA.XTH23.b,* together with *BolC.XTH11.a* and *BolC.XTH11.b*, showed different expression patterns, suggesting that divergence in gene expression may have been associated with the acquisition of novel characteristics (Fig. 5, Additional file 8). *BraA.XTH5.a* was expressed in roots but *BraA.XTH5.b* was not expressed in any of the tissues tested, suggesting that it had experienced nonfunctionalization after the duplication events (Fig. 5a, Additional file 8).

## Conclusions

In this study, 53 and 38 *XTH* genes were identified in *B. rapa* and *B. oleracea* respectively. They, together with the 33 Arabidopsis *XTH* genes, were classified into three groups (Early diverging group, Group I/II and Group III) by phylogenetic analysis based on clade support values, the topology of the phylogenetic tree, and the previous classification of XTH families in Arabidopsis. Exon-intron distribution and comparisons of conserved motifs also supported this classification of *XTH* genes. Analysis of expansion mechanisms revealed that a WGT event exerted the most major influence, followed by TD events, on the expansion of the *XTH* gene family in both *Brassica* species. Gene loss events have occurred in the *XTH* gene family in the two species; the extent of loss was greater in *B. oleracea* than in *B. rapa*. RNA-seq data analysis provided insight into species-specific functional divergence among members of the *XTH* gene family. Taken together, these results increase our understanding of the evolution of the *XTH* gene family and provide a reference for future determination of the functions of each *XTH* gene across *Brassica* species.

## Methods

### Data sources

Genomic sequences, CDS sequences, protein sequences and annotation information for *B. rapa* and *B. oleracea* were downloaded from the BRAD database (http://brassicadb.org) [58]. *A. thaliana* XTH protein sequences were downloaded from TAIR (http://www.arabidopsis.org/) [59].

Identification of XTH genes and analysis of their characteristics

Three methods were used to identify XTH proteins in this study. First, all 33 *A. thaliana* XTH sequences obtained from the TAIR database were used as query sequences to carry out a BLASTp (E-value <1e-5) search for all protein sequences from *B. rapa* and *B. oleracea* in the BRAD database. Second, the HMM profiles of the Glyco_hydro_16 domain (PF00722) and XET_C domain (PF06955) were obtained from the Pfam database (https://Pfam.xfam.org/) [60] and used to search all *B. rapa* and *B. oleracea* proteins with the HMM search tool in the TBtools software package [58] with default parameters. The third approach was to search for syntenic genes in the BRAD database by inputting the *A. thaliana* XTH gene IDs [61]. After integrating the results of the three methods, all redundant sequences were removed manually, after which candidate XTH protein sequences were filtered using the CDD tool (http://www.ncbi.nlm.nih.gov/Structure/cdd/wrpsb.cgi/) [62]. Only proteins that contained both the Glyco_hydro_16 domain and the XET_C domain were regarded as XTHs and reserved for further analysis. Finally, all genes identified as encoding XTH proteins were designated with reference to a previous study [63].

The molecular weight (Mw), number of amino acids and theoretical isoelectric point (pI) of each protein were obtained from ProtParam (http://web.expasy.org/protparam/) [64]. Plant-mPLoc (http://www.csbio.sjtu.edu.cn/bioinf/plant-multi/) [65] was used to predict patterns of protein subcellular localization. The TargetP-2.0 Server (http://www.cbs.dtu.dk/services/TargetP/) [66] was used to predict the presence or absence of signal peptides. The MEME tool (http://meme-suite.org/tools/meme) [67] was employed to predict and analyze the motifs in each protein, with parameters set as follows: motif width 6–60, maximum number of motifs 10 and default values were used for the remaining parameters. The distribution of motifs was illustrated using the Redraw motif pattern tool in TBtools.

### Comparative phylogenetic analysis of XTH proteins

Multiple sequence alignments of the full-length XTH protein sequences from *B. rapa*, *B. oleracea* and *A. thaliana* were performed using Clustal X1.8, then MEGA7 was used to analyze the results (Additional file 9) [68]. The phylogenetic tree was constructed based on the Maximum Likelihood (ML) method, with the JTT + G model and 1000 bootstrap replicates. The phylogenetic tree was visualized using the iTOL online tool (https://itol.embl.de/) [69].

### Structural-based sequence alignment

ESPript (http://espript.ibcp.fr/ESPript/ESPript/) [70] was used to predict the secondary structures as well as the presence of structural elements in the XTH protein sequences. The crystal structure of PttXET16 (PDB id:1UN1) [53] was obtained from the PDB databank to locate secondary structures.

### Analysis of chromosomal locations and gene duplication

The chromosomal locations of *XTH* genes were derived from BRAD. All *XTH* genes were mapped to chromosomes by MapChart [71] except for a few that were located on unassigned scaffolds. Duplicated *XTH* gene pairs were identified by the BLASTn program with both coverage and identity set to > 80% in the two species [72]. Putative tandemly-duplicated genes in *A. thaliana*, *B. rapa*, and *B. oleracea* were retrieved from PTGBase (http://ocri-genomics.org/PTGBase) [73].

To estimate the modes of selection acting on *XTH* genes, the Ka/Ks ratios between duplicated *XTH* gene pairs were calculated. Ka/Ks ratios greater than 1, less than 1, and equal to 1 represented positive selection, negative selection, and neutral selection respectively [74]. For each gene pair, the Ks value was used to estimate the divergence time (T) as millions of years ago based on a rate of 1.5 × 10^− 8^ substitutions per site per year, using the formula T = Ks/(2 × 1.5 × 10^− 8^) Mya [75].

### Analysis of tissue expression patterns

RNA-seq expression data for *B. rapa* tissues (GSE43245) and *B. oleracea* tissues (GSE42891) were downloaded from the GEO database at NCBI (http://www.ncbi.nlm.nih.gov/geo/) [41, 76]. The XTH expression data were measured using the expressed FPKM values for transcripts assembled and analyzed in a previous study [77, 78]. FPKM values for different tissue were subjected to hierarchical clustering analysis with TBtools. The data were normalized in order to more intuitively examine differences in expression of the same gene in different samples and represented as a heatmap with TBtools; genes with FPKM values of less than one in all samples were not included in the heatmap.

### Ethics approval

This article does not contain any studies with human participants or animals performed by any of the authors.

## Supplementary Information

**Additional file 1. **XTHs in *B. rapa* and *B. oleracea* with their Arabidopsis orthologs.

**Additional file 2.** The best-hits between BraXTHs in this study and BraXTHs identified in previous report.

**Additional file 3.** The ML resulting phylogeny in Nexus format.

**Additional file 4. **Structure-based sequence alignment of BraXTHs and PttXET16A. Sequences were aligned using ClustalX and generated by ESPript [70]. The secondary structure elements indicated above the alignment are those of PttXET16A, *Populus tremula* × *tremuloides* XET16A (AF515607), whose structure has been experimentally determined [45]. Blue frames indicate conserved residues, white letters in red boxes indicate strict identity, and red letters in white boxes indicate similarity. The predicted α-helices and β-strands are represented by spirals and horizontal arrows, respectively.

**Additional file 5. **Structure-based sequence alignment of BolXTHs and PttXET16A. Sequences were aligned using ClustalX and generated by ESPript [70]. The secondary structure elements indicated above the alignment are those of PttXET16A, *Populus tremula* × *tremuloides* XET16A (AF515607), whose structure has been experimentally determined [45]. Blue frames indicate conserved residues, white letters in red boxes indicate strict identity, and red letters in white boxes indicate similarity. The predicted α-helices and β-strands are represented by spirals and horizontal arrows, respectively.

**Additional file 6. ***XTH* genes syntenic in *A. thaliana*, *B. rapa* and *B. oleracea*.

**Additional file 7. **Ka/Ks analysis and estimated time of divergence of duplicated pairs of XTH genes in *B. rapa* and *B. oleracea*.

**Additional file 8. **FPKM values for *XTH* genes in different tissues of *B. rapa* and *B. oleracea*.

**Additional file 9.** The alignment file used as input for the ML analysis in FASTA format.

## Data Availability

All data generated or analyzed during this study are included in this published article and the additional files.

## Abbreviations

*A. thaliana*: *Arabidopsis thaliana*
*B. oleracea*: *Brassica oleracea*
*B. rapa*: *Brassica rapa*
BLASTp: Basic Local Alignment Search Tool
FPKM: Fragments per kilobase of transcript per million fragments sequenced
Ka: Non-synonymous substitution
Ks: Synonymous substitution
MW: Molecular weight
MYA: Million years ago
NJ: Neighbor-joining
XTH: Xyloglucan endotransglucosylase/hydrolase
LF: Least fractionated
MF1: Medium fractionated
MF2: Most fractionated
WGD: Whole genome duplication
WGT: Whole genome triplication
TD: Tandem duplication
HMM: Hidden Markov Model
