# Supplementary material for: Genome-wide identification, and phylogenetic and expression profiling analyses, of XTH gene families in Brassica rapa L. and Brassica oleracea L
Source: BMC Genomics. 2020 Nov 11;21:782. doi: 10.1186/s12864-020-07153-1 (PMC7656703; doi:10.1186/s12864-020-07153-1)
Supplement: Supplementary file 3 — Additional file 3. The ML resulting phylogeny in Nexus format. [file 12864_2020_7153_MOESM3_ESM.docx]

((((((((((((((((((BraA.XTH17.c,BolC.XTH20)0.9700,(BraA.XTH17.d,BolC.XTH17.b)0.9400)0.4200,(BraA.XTH17.a,(BraA.XTH17.b,BolC.XTH17.a)0.8400)0.9000)0.4700,(BraA.XTH17.e,BraA.XTH18)0.8900)0.4300,AtXTH20)0.5400,(AtXTH17,(AtXTH18,AtXTH19)0.4400)0.8200)0.9700,(AtXTH25,(BraA.XTH25.a,(BraA.XTH25.b,BolC.XTH25)0.9900)0.5900)1.0000)0.3100,((((BraA.XTH23.a,BolC.XTH23)0.4600,BraA.XTH23.b)0.9100,AtXTH23)0.9800,(((BraA.XTH22.b,BolC.XTH22.a)0.6500,AtXTH22)0.7300,(BraA.XTH22.d,(BraA.XTH22.a,BraA.XTH22.c)0.3500)0.5100)0.8500)0.6900)0.3200,(BolC.XTH24.c,(((BraA.XTH24.a,BolC.XTH24.d)0.8000,AtXTH24)0.3700,((BraA.XTH24.c,BolC.XTH24.b)1.0000,(BraA.XTH24.b,BolC.XTH24.a)0.5900)0.3200)0.5300)0.9900)0.4700,(AtXTH21,(BraA.XTH21,BolC.XTH21)0.9600)0.9900)0.4800,(BraA.XTH22.e,BolC.XTH22.b)1.0000)0.7000,((((BraA.XTH15,BolC.XTH15)0.9600,AtXTH15)0.8100,(AtXTH16,(BraA.XTH16,BolC.XTH16)0.9500)0.8100)1.0000,(AtXTH12,(((BraA.XTH14.b,BraA.XTH14.a)0.6100,AtXTH14)0.8100,(((BraA.XTH12.c,BolC.XTH12)0.8000,AtXTH13)0.5500,(BraA.XTH12.b,(BraA.XTH12.a,BolC.XTH13)0.6000)0.4600)0.5700)0.4500)0.9200)0.4400)0.6800,(AtXTH26,(BraA.XTH26,BolC.XTH26)0.7400)1.0000)0.5400,(((BraA.XTH4,BolC.XTH4)0.6400,AtXTH4)0.9900,(AtXTH5,(BraA.XTH5.a,(BraA.XTH5.b,BolC.XTH5)0.7500)0.4000)0.5700)0.8900)0.2800,(((BraA.XTH8,BolC.XTH8)0.7800,AtXTH8)1.0000,(((((BraA.XTH9.a,BolC.XTH9.a)0.9600,BolC.XTH9.b)0.4200,BraA.XTH9.b)0.3900,AtXTH9)1.0000,((AtXTH6,BraA.XTH6)0.8100,(AtXTH7,(BraA.XTH7,BolC.XTH7)0.9800)0.9200)0.9900)0.3600)0.1800)0.2200,(AtXTH10,BraA.XTH10)1.0000)0.8200,(((((((BolC.XTH32.b,BolC.XTH32.c)0.6500,BraA.XTH32.a)0.1900,(BraA.XTH32.b,BolC.XTH32.a)0.8000)0.3600,BraA.XTH32.c)0.3200,AtXTH32)0.9900,((BraA.XTH31.a,BolC.XTH31)0.8000,(AtXTH31,BraA.XTH31.b)0.4600)0.8900)1.0000,(((BraA.XTH33,BolC.XTH33)0.7600,AtXTH33)1.0000,(((((BraA.XTH27.a,BolC.XTH27.b)0.9300,AtXTH27)0.4100,(BraA.XTH27.b,BolC.XTH27.a)0.9600)0.6400,(AtXTH28,(BraA.XTH28,BolC.XTH28)0.9900)0.9400)0.9800,(((BraA.XTH30,BolC.XTH30.b)0.6600,(BolC.XTH30.a,AtXTH30)0.7600)0.5500,(AtXTH29,(BraA.XTH29.a,(BolC.XTH29.b,(BraA.XTH29.b,BolC.XTH29.a)0.5100)0.3200)0.5700)0.9800)0.8800)0.8100)0.7800)0.9100)0.3100,(((((BraA.XTH2.b,BolC.XTH2)0.9400,BraA.XTH2.a)0.9700,AtXTH2)0.3600,AtXTH1)0.6600,((AtXTH3,BraA.XTH3)0.9400,(AtXTH11,(BraA.XTH11.a,(BraA.XTH11.b,(BolC.XTH11.b,BolC.XTH11.a)0.4200)0.9800)0.6200)1.0000)0.5200)0.7600,1GBG);
