# Supplementary material for: Genome-wide identification, and phylogenetic and expression profiling analyses, of XTH gene families in Brassica rapa L. and Brassica oleracea L
Source: BMC Genomics. 2020 Nov 11;21:782. doi: 10.1186/s12864-020-07153-1 (PMC7656703; doi:10.1186/s12864-020-07153-1)
Supplement: Supplementary file 4 — Additional file 4. Structure-based sequence alignment of BraXTHs and PttXET16A. Sequences were aligned using ClustalX and generated by ESPript [70]. The secondary structure elements indicated above the alignment are those of PttXET16A, Populus tremula × tremuloides XET16A (AF515607), whose structure has been experimentally determined [45]. Blue frames indicate conserved residues, white letters in red boxes indicate strict identity, and red letters in white boxes indicate similarity. The predicted α-helices and β-strands are represented by spirals and horizontal arrows, respectively. [file 12864_2020_7153_MOESM4_ESM.pdf]

PttXET16A

```
                         1        10        20        30
PttXET16A     ...........MAAAYPWTLFLGMLVMVSGTMGAALRKPVD.............................
BraA.XTH9.a   ................MVGMSWFMCMMMMVCVISCGEAAPG.............................
BraA.XTH9.b   ............................MVCVVSCGEAAPG.............................
BraA.XTH5.a   ............MGRLPLTFCLTFLLMATVTFGVPPKKSID.............................
BraA.XTH5.b   ..........................MATVTFGVPPKKAVD.............................
BraA.XTH4     ..........MAVSSTPWALVALFLMASSTVMAIPPRKAID.............................
BraA.XTH8     ............MEKRYSSMAAVLFFVAALMVSSSITAVPT.............................
BraA.XTH7     ...........MVVSLFSARNAFFISLCLFAALYRPVLSKP.............................
BraA.XTH6     ................MMAETPFLCIVTLCTLMFIQISARP.............................
BraA.XTH22.c  ......................................................................
BraA.XTH22.d  ......................................................................
BraA.XTH22.a  .................MAHSYFLPL.FISLIVISSVSAN..............................
BraA.XTH22.b  .................MANSYLFPL.FLSLIVISSVSAN..............................
BraA.XTH23.a  ..............MAMINYSTILSP.LLAAIMIYSVSAN..............................
BraA.XTH23.b  ..............MAMINYSTILSP.LLAAIMIYSVSAN..............................
BraA.XTH24.a  ................MSPFKIFLFS.ALLAAAFLASAAD..............................
BraA.XTH24.b  ................MSPFKIFFFA.ALLAAVFSFSAAD..............................
BraA.XTH24.c  ......................................................................
BraA.XTH17.e  ............MKFSCGTRFAFLVL.FLFAAQSVAVYAG..............................
BraA.XTH18    ............MKLSCGTRFTFLVL.FLFAAQSVAVYAG..............................
BraA.XTH17.b  ............MKFSCGTRFAFLVL.FLIAAQSVAVYAG..............................
BraA.XTH17.a  ............MKFSCGTRFAFLAL.FLFALQSVCVYAG..............................
BraA.XTH17.d  ............MKSSCGTRFAFLVL.FLFAVQSVCVNAGT.............................
BraA.XTH17.c  ............MKSSCGTRFTFLAL.YLFALQ..CVYAG..............................
BraA.XTH25.a  ...........MDRHSTLILSILLN..ALTTTFFSPVYAG..............................
BraA.XTH25.b  ...........MDR.STFILSILLTLTATTTTFFSPVYAG..............................
BraA.XTH16    ..............MGQFLNLT.VLVTVLVLTTVGTAYSG..............................
BraA.XTH15    ..............MGQSSSFTTVMVAVLLVMMFGSAYSG..............................
BraA.XTH12.a  .............MAALATKQSLLLLLSSL.LLLIGVSTG..............................
BraA.XTH12.c  .............MAAFATKQSMLLLSLSL.LLLIGVSTG..............................
BraA.XTH12.b  .............MAGFETK...LMLTLSL.LLLIGVCTG..............................
BraA.XTH14.b  .............MAVSATKKPLLLLSLFF..FVVAASAG..............................
BraA.XTH14.a  .............MAVPASKKPLLLSFLWFGLFVAAASAG..............................
BraA.XTH22.e  ..................MGCSPILISLFILSCSALILAG..............................
BraA.XTH21    ......................................................................
BraA.XTH26    ............MAGLRVQTLIFVLVGALAILDRTFVEAN..............................
BraA.XTH10    ........MTSVKRSKSFVLLIYLISSLLLRVSEASVVSS..............................
BraA.XTH32.a  .MTNSLISLLPIFHLLVLLGSSVNAYWPPSPGYWPSSKVG..............................
BraA.XTH32.c  .MGSSLISLLPIFHLLVLLGSSVNAYWPPSPGYWPSSKVV..............................
BraA.XTH32.b  .MGNSLISLLPVFQFLVLLGSSVNAYWPPSPGYWPSSKVG..............................
BraA.XTH31.a  ......MAFPLILLAFVVLCSSGYSQRSPSPGYYPSSRVP..............................
BraA.XTH31.b  ......MAFLVILLALLVFSHCGYSQRSPSPGYYPSSRVP..............................
BraA.XTH29.a  ...MSD......LKYRLKVMVM.MMAIVSWRCVLGLENIN..............................
BraA.XTH29.b  ...MRDSIYLLWINHGLKVMMV.MMMFVSWRGVLGLENIN..............................
BraA.XTH30    ...MSK......LSYNLIFFIV.FLCLGLRSSAF..TNLN..............................
BraA.XTH27.b  .......................MSLFSGFVSGFTLQNLP..............................
BraA.XTH27.a  ............METLSRFLVF.MSLFSGLGSGFTLQNLP..............................
BraA.XTH28    ............MELLARFLAF.MSLFTSLVSGFALQKLP..............................
BraA.XTH33    MASLKNYNMKILLETAVVFCLCSFSLVSSHSRKFTTPNVTRVTDQF........................
BraA.XTH2.b   ...............MFSILDFVLVLLV.IATVD..ASVPG.............................
BraA.XTH2.a   .............MDKREYMFSVLVLFL.IGTVG..AGVPA.............................
BraA.XTH3     .......MNIFRLAGDMTHLASVLVLLLKIHTIKSCAGVSLRTQELYAIVFATRYLDIFTSFVSVYNTFM
BraA.XTH11.a  ............MKMSGSDEKILLLIGMVVLTVAVRAIDEDPT...........................
BraA.XTH11.b  ............MKMRGSDQKNILIVMMVVIVAAT.ARGED.............................
```

PttXET16A

```
PttXET16A     ......................................................................
BraA.XTH9.a   ......................................................................
BraA.XTH9.b   ......................................................................
BraA.XTH5.a   ......................................................................
BraA.XTH5.b   ......................................................................
BraA.XTH4     ......................................................................
BraA.XTH8     ......................................................................
BraA.XTH7     ......................................................................
BraA.XTH6     ......................................................................
BraA.XTH22.c  ......................................................................
BraA.XTH22.d  ......................................................................
BraA.XTH22.a  ......................................................................
BraA.XTH22.b  ......................................................................
BraA.XTH23.a  ......................................................................
BraA.XTH23.b  ......................................................................
BraA.XTH24.a  ......................................................................
BraA.XTH24.b  ......................................................................
BraA.XTH24.c  ......................................................................
BraA.XTH17.e  ......................................................................
BraA.XTH18    ......................................................................
BraA.XTH17.b  ......................................................................
BraA.XTH17.a  ......................................................................
BraA.XTH17.d  ......................................................................
BraA.XTH17.c  ......................................................................
BraA.XTH25.a  ......................................................................
BraA.XTH25.b  ......................................................................
BraA.XTH16    ......................................................................
BraA.XTH15    ......................................................................
BraA.XTH12.a  ......................................................................
BraA.XTH12.c  ......................................................................
BraA.XTH12.b  ......................................................................
BraA.XTH14.b  ......................................................................
BraA.XTH14.a  ......................................................................
BraA.XTH22.e  ......................................................................
BraA.XTH21    ......................................................................
BraA.XTH26    ......................................................................
BraA.XTH10    ......................................................................
BraA.XTH32.a  ......................................................................
BraA.XTH32.c  ......................................................................
BraA.XTH32.b  ......................................................................
BraA.XTH31.a  ......................................................................
BraA.XTH31.b  ......................................................................
BraA.XTH29.a  ......................................................................
BraA.XTH29.b  ......................................................................
BraA.XTH30    ......................................................................
BraA.XTH27.b  ......................................................................
BraA.XTH27.a  ......................................................................
BraA.XTH28    ......................................................................
BraA.XTH33    ......................................................................
BraA.XTH2.b   ......................................................................
BraA.XTH2.a   ......................................................................
BraA.XTH3     KLVFLGSSFSIVWYMRYHKAVHRTYDREQDTFRHWFLVLPCLVLALLIHEKFTFLEVLWTFSLYLEAVAI
BraA.XTH11.a  ......................................................................
BraA.XTH11.b  ......................................................................
```

PttXET16A

```
PttXET16A    ......................................................................
BraA.XTH9.a  ......................................................................
BraA.XTH9.b  ......................................................................
BraA.XTH5.a  ......................................................................
BraA.XTH5.b  ......................................................................
BraA.XTH4    ......................................................................
BraA.XTH8    ......................................................................
BraA.XTH7    ......................................................................
BraA.XTH6    ......................................................................
BraA.XTH22.c ......................................................................
BraA.XTH22.d ......................................................................
BraA.XTH22.a ......................................................................
BraA.XTH22.b ......................................................................
BraA.XTH23.a ......................................................................
BraA.XTH23.b ......................................................................
BraA.XTH24.a ......................................................................
BraA.XTH24.b ......................................................................
BraA.XTH24.c ......................................................................
BraA.XTH17.e ......................................................................
BraA.XTH18   ......................................................................
BraA.XTH17.b ......................................................................
BraA.XTH17.a ......................................................................
BraA.XTH17.d ......................................................................
BraA.XTH17.c ......................................................................
BraA.XTH25.a ......................................................................
BraA.XTH25.b ......................................................................
BraA.XTH16   ......................................................................
BraA.XTH15   ......................................................................
BraA.XTH12.a ......................................................................
BraA.XTH12.c ......................................................................
BraA.XTH12.b ......................................................................
BraA.XTH14.b ......................................................................
BraA.XTH14.a ......................................................................
BraA.XTH22.e ......................................................................
BraA.XTH21   ......................................................................
BraA.XTH26   ......................................................................
BraA.XTH10   ......................................................................
BraA.XTH32.a ......................................................................
BraA.XTH32.c ......................................................................
BraA.XTH32.b ......................................................................
BraA.XTH31.a ......................................................................
BraA.XTH31.b ......................................................................
BraA.XTH29.a ......................................................................
BraA.XTH29.b ......................................................................
BraA.XTH30   ......................................................................
BraA.XTH27.b ......................................................................
BraA.XTH27.a ......................................................................
BraA.XTH28   ......................................................................
BraA.XTH33   ......................................................................
BraA.XTH2.b  ........................GGG...........................................
BraA.XTH2.a  ........................GGRP..........................................
BraA.XTH3    LPQLVLLQRTRNIDNLTGQYIFLLGGYRGLYILNWIYRYFTEPHFVHWITWIAGLVQTLLYADFFYYYFL
BraA.XTH11.a ......................................................................
BraA.XTH11.b ......................................................................
```

PttXET16A

```
                            α1     β1      η1   β2   η2    β3         β4          β5
                                   40          50          60         70          80
PttXET16A    ...........VAFGRNYVPTWAFDH.IKYFNGGNEIQLHLDKYTGTGFQSKGSYLFGHFSMQMKLVPGD
BraA.XTH9.a  ...........AKFEDLYRSSWAMDH.CVN..DGEVTKLKLDNSSGAGFESRSKYLFGKVSIQIKLVEGD
BraA.XTH9.b  ...........AKFEELYRSSWAMDH.CVN..DGEVTKLKLDNSSGAGFESRSKYLFGKVSIQIKLVEGD
BraA.XTH5.a  ...........VPFGRNYFPTWAFDH.IKYLNGGSEVHLVLDKHTGTGFQSKGSYLFGHFSMHIKMVPGD
BraA.XTH5.b  ...........VPFGRNYFPTWAFDH.IKYLNGGSEVHLFLDKYTGTGFQSKGSYLFGHFSMHIKMVPGD
BraA.XTH4    ...........VPFGRNYVPTWAFDH.QKQLNGGSELQLILDKYTGTGFQSKGSYLFGHFSMHIKLPAGD
BraA.XTH8    ...........QSFEENFNIMWSENH.FTTSEDGQIWNLALDNDTGCGFQTKHMYRFGWFSMKLKLVGGD
BraA.XTH7    ...........AKFADDFRITWSDTH.ITQIDGGRAIQLKLDPSSGCGFASKKQYLFGRVSMKIKLIPGD
BraA.XTH6    ...........TTFAEDFKAAWSESH.IRQVDGGKAIQLVLDQSTGCGFSSKRKYLFGKVSMKIKLIPGD
BraA.XTH22.c .............................................................MQMKLVPGN
BraA.XTH22.d .............................................................MQMKLVPGN
BraA.XTH22.a .............FQRDVEITWGDGRGQITNN.GELLTLSLDKSSGSGFQSKNEYLFGKIDMQMKLVPGN
BraA.XTH22.b .............FQRDVEITWGDGRGQITNN.GELLTLSLDKSSGSGFQSKNEYLFGKIDMQMKLVPGN
BraA.XTH23.a .............FHQDVEITWGDGRGQITNN.GDLLTLSLDKSSGSGFQSKNEYLFGKIDMQIKLVAGN
BraA.XTH23.b .............FHQDVEITWGDGRGQITNN.GDLLTLSLDKSSGSGFQSKNEYLFGKIDMQIKLVAGN
BraA.XTH24.a .............FNSDVNVAWGNGRGKILNN.GQLLTLTLDKSSGSGFQSKTEYLFGKIDMQIKLVPGN
BraA.XTH24.b .............FNSDVNVAWGNGRGKILNN.GQLLTLSLDRSSGSGFQSKTEYLFGKIDMQIKLVPGN
BraA.XTH24.c .............................................................MQIKLVPGN
BraA.XTH17.e ............SFHKDVKIHWGDGRGKIRDRDGKLLSLSLDKSSGSGFQSNQEFLYGKAEVQMKLVPGN
BraA.XTH18   ............SFHKDVQIHWGDGRGKVRDRDGKLLSLSLDKSSGSGFQSHQEFLYGKAEVQMKLVPGN
BraA.XTH17.b ............SFHKDVQIHWGDGRGKVRDRDGKLLSLSLDKSSGSGFQSNQEFLYGKAEVQMKLVAGN
BraA.XTH17.a ............SFHKDVQIHWGDGRGKIHDNEGKLLSLSLDKSSGSGFQSNQEFLYGKAEVQMKLVPGN
BraA.XTH17.d ...........GSFHKDVKIHWGDGRGKIHDNEGKLLSLSLDKSSGSGFQSNDEFLYGKAEVQMKLVPGN
BraA.XTH17.c ............SFHKDVNIHWGNGRGKIHDNEGKLLSLSLDKSSGSGFQSNQEFLYGKAEVQMKLVPGN
BraA.XTH25.a ............TFDREFDITWGGGRGKVLNN.GELLTLSLDRASGSGFQSKKESLFGKIDMQIKLVPGN
BraA.XTH25.b ............TFDMEFDITWGDGRGKVLNN.GELLTLSLDRASGSGFQSKKEYLFGKIDMQLKLVPGN
BraA.XTH16   ............SFNEEFDLTWGEHRGKIFSG.GKMLSLSLDRVSGSGFKSKKEYLFGRIDMQLKLVAGN
BraA.XTH15   ............NFFNEFDLTWGDHRGKIFNG.GNMLSLSLDRVSGSGFKSKKEYLFGRIDMQLKLVAGN
BraA.XTH12.a ............SFYDNFDITWGNGRANIFES.GQLLTCTLDKISGSGFQSKKEYLFGKIDMKMKLVAGN
BraA.XTH12.c ............SFYDNFDITWGNGRANIFES.GQLLTCTLDKISGSGFQSKKEDLFGKIDMKMKLVAGN
BraA.XTH12.b ............SFYDNFDITWGDGRANIFES.GQLLSCTLDKISGSGFQSKKEYLFGKIDMKMKLVAGN
BraA.XTH14.b ............NFYESFDITWGDGRAKILED.GQLLTCTLDKISGSGFQSKKEYLYGKIDMKLKLVAGN
BraA.XTH14.a ............SFYESFDITWGDGRADIYET.GQLLTCTLDKVSGSGFQSKKEYLYGKIDMKLKLVAGN
BraA.XTH22.e ............DFRKDIDIVWGSDKAKILEN.GQAITLSLDKASGSGFESKAQFLFGKVSMELKLVPGN
BraA.XTH21   .............................................................MQIKLVPGN
BraA.XTH26   .............FDKNFIVTWGKHHIGTTNG...NLRLVLDKSAGSAIRSKVAHLFGTVEMLIKLVPGN
BraA.XTH10   ...........GDFNKDFLVTWSPTHVNTSSD.GRSRTLKLDQESGASFSSIQTFLFGKIDMKIKLIPGP
BraA.XTH32.a ..........SLNFYKGFRNLWGPQHQRMD...QNALTIWLDRTSGSGFKSVKPFRSGYFGASIKLQPGY
BraA.XTH32.c ..........SLSFYKGFRNLWGPQHQRMD...QNALTIWLDRVSGSGFKSVKPFRSGYFGASIKLQPGY
BraA.XTH32.b ..........SLSFYKGFKNLWGPQHQRTD...QNGLTIWLDRTSGSGFKSVKPFRSGYFGASIKLQPGY
BraA.XTH31.a ..........TSPFDRDFRTLWGSQHQRTE...QDVITLLLDKSSGSGFKSLRSYKSGYFGASIKLQSGY
BraA.XTH31.b ..........TSPYDRDFRTQWGFQHQRRE...HDITTLWLDKASGSGFKSIRPYISGYFGASIKLQSGY
BraA.XTH29.a ..........PIFFDEGLSHLFGESNLIRSPD.DRSVRLLLDKYTGSGFISSSMYQHGFFSSLIKLPGAN
BraA.XTH29.b ..........PIFFDEGLSHLFGESNLIRSPD.DRSVRLLLDKYTGSGFISSNMYQHGFFSSLIKLPGAY
BraA.XTH30   ..........TLSFEESLSPLFGDGNLVRSPD.DLSVRLLLDKYTGSGFISSNMYQHGFYSSMIKLPADY
BraA.XTH27.b ..........VTSFEESYTQLFGDKNLFVHKD.GKTVRLTLDERTGSGFVSNDLYLHGLFSASIKLPSDY
BraA.XTH27.a ..........ITSFEESYMQLFGDKNLFVHKD.GKSVRLTLDERTGSGFVSNDLYLHGFFSASIKLPSDY
BraA.XTH28   ..........LIQFDEGYTHLFGDQNLIVHRD.GKSVRLILDERTGSGFVSNDIYLHGFFSSSIKLPADY
BraA.XTH33   .........SRIAIESGFSKRFGDHNIIIN...GSLAKLTLDKSSGAGLVTKNTYHYGFFSARLKLPAGF
BraA.XTH2.b  .............FDENYVVTWGN...VLKLNQG.EVQLSMDKSAGSAFESKHKFGSGFFQMRIKLPPKD
BraA.XTH2.a  .............FDDNYVVTWGN...VLKLNQGREVQLSMDKSSGAGFQSKHKFGSGFFQMRIKLPPKD
BraA.XTH3    RFIGGRGVEKYVTFGQNYVVKWGQGH.ISTLHSGKEVDLYMDQSSGAGFESKNIYGSGLFQMRIKVPGGN
BraA.XTH11.a ..........GFVTWGHNYYKTWGQP..ALVINETSELHLTLDHKSGSGFESNSIYGSGSFNMRIKAPQTK
BraA.XTH11.b ..........GFVTWGDNYYTTWGHP..ALVINETSELQLTLDHKSGSGFESYKIYGSGSFNIRIKAPQSK
```

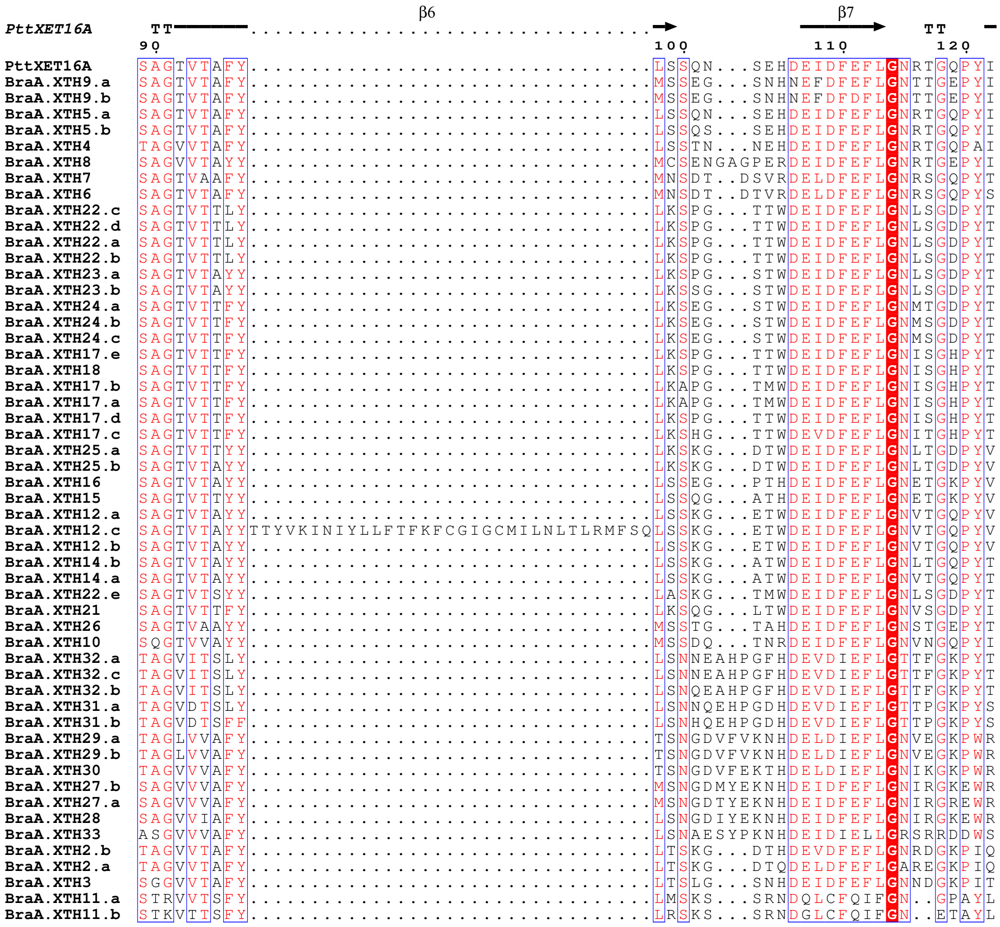
PttXET16A
TT
β6
β7
TT
90
100
110
120
PttXET16A
BraA.XTH9.a
BraA.XTH9.b
BraA.XTH5.a
BraA.XTH5.b
BraA.XTH4
BraA.XTH8
BraA.XTH7
BraA.XTH6
BraA.XTH22.c
BraA.XTH22.d
BraA.XTH22.a
BraA.XTH22.b
BraA.XTH23.a
BraA.XTH23.b
BraA.XTH24.a
BraA.XTH24.b
BraA.XTH24.c
BraA.XTH17.e
BraA.XTH18
BraA.XTH17.b
BraA.XTH17.a
BraA.XTH17.d
BraA.XTH17.c
BraA.XTH25.a
BraA.XTH25.b
BraA.XTH16
BraA.XTH15
BraA.XTH12.a
BraA.XTH12.c
BraA.XTH12.b
BraA.XTH14.b
BraA.XTH14.a
BraA.XTH22.e
BraA.XTH21
BraA.XTH26
BraA.XTH10
BraA.XTH32.a
BraA.XTH32.c
BraA.XTH32.b
BraA.XTH31.a
BraA.XTH31.b
BraA.XTH29.a
BraA.XTH29.b
BraA.XTH30
BraA.XTH27.b
BraA.XTH27.a
BraA.XTH28
BraA.XTH33
BraA.XTH2.b
BraA.XTH2.a
BraA.XTH3
BraA.XTH11.a
BraA.XTH11.b

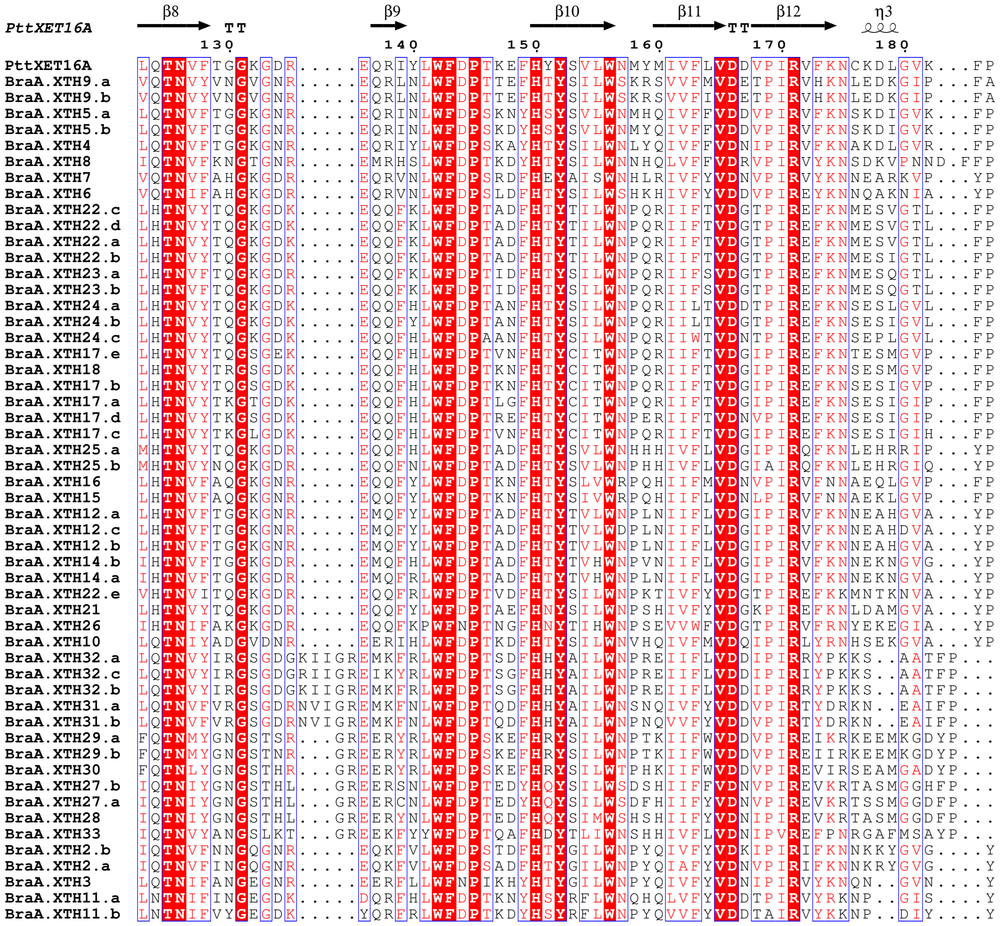
PttXET16A
β8
TT
β9
β10
β11
TT
β12
η3
130
140
150
160
170
180
PttXET16A
BraA.XTH9.a
BraA.XTH9.b
BraA.XTH5.a
BraA.XTH5.b
BraA.XTH4
BraA.XTH8
BraA.XTH7
BraA.XTH6
BraA.XTH22.c
BraA.XTH22.d
BraA.XTH22.a
BraA.XTH22.b
BraA.XTH23.a
BraA.XTH23.b
BraA.XTH24.a
BraA.XTH24.b
BraA.XTH24.c
BraA.XTH17.e
BraA.XTH18
BraA.XTH17.b
BraA.XTH17.a
BraA.XTH17.d
BraA.XTH17.c
BraA.XTH25.a
BraA.XTH25.b
BraA.XTH16
BraA.XTH15
BraA.XTH12.a
BraA.XTH12.c
BraA.XTH12.b
BraA.XTH14.b
BraA.XTH14.a
BraA.XTH22.e
BraA.XTH21
BraA.XTH26
BraA.XTH10
BraA.XTH32.a
BraA.XTH32.c
BraA.XTH32.b
BraA.XTH31.a
BraA.XTH31.b
BraA.XTH29.a
BraA.XTH29.b
BraA.XTH30
BraA.XTH27.b
BraA.XTH27.a
BraA.XTH28
BraA.XTH33
BraA.XTH2.b
BraA.XTH2.a
BraA.XTH3
BraA.XTH11.a
BraA.XTH11.b

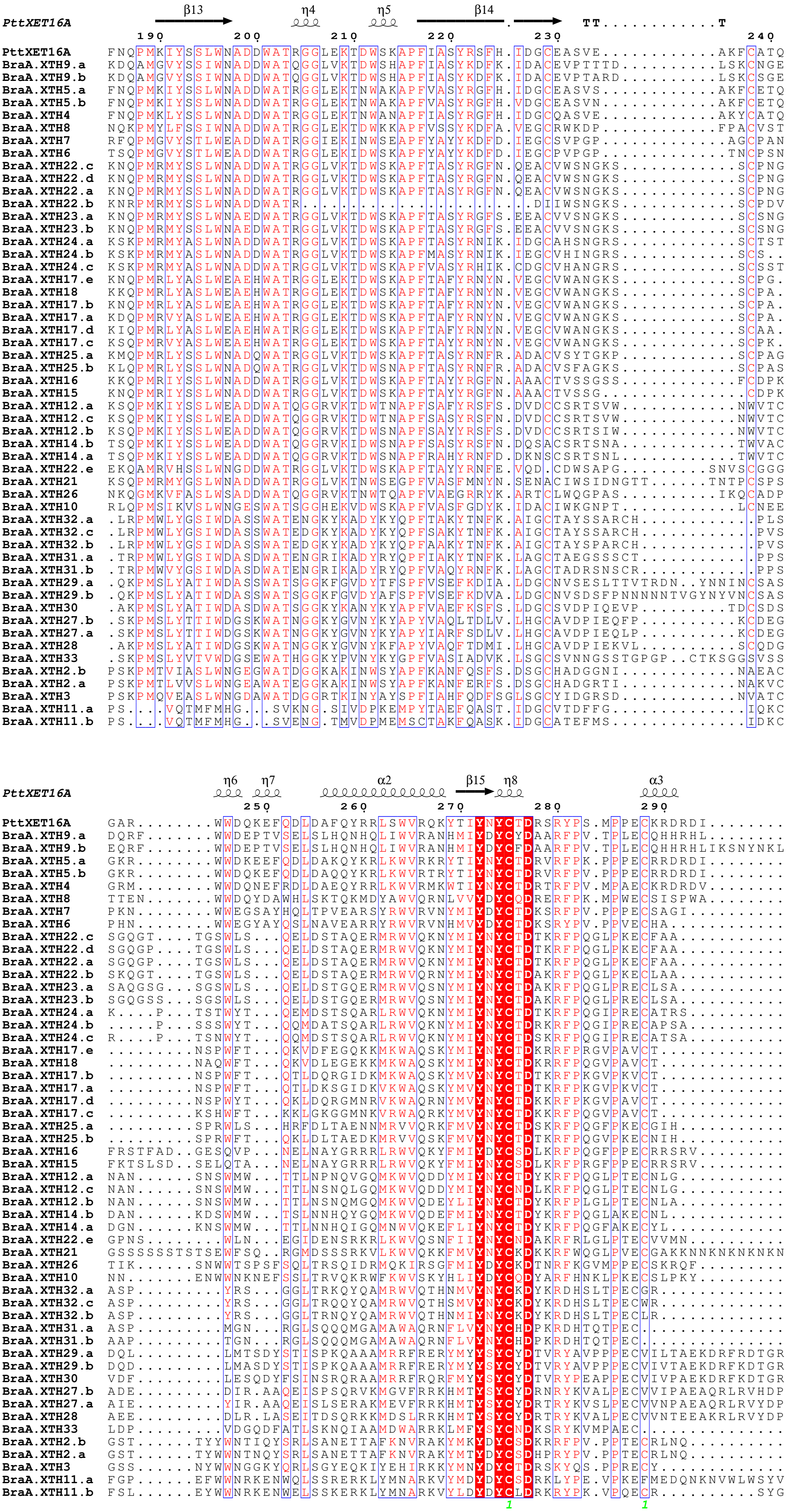

PttXET16A
β13 η4 η5 β14 TT...........T
190 200 210 220 230 240
PttXET16A FNQPMKIYSSLWNADDWATRGGLEKTDWSKAPFIASYRSFH.IDGCEASVE...........AKFCATQ
BraA.XTH9.a KDQAMGVYSSIWNADDWATQGGLVKTDWSHAPFIASYKDFK.IDACEVPTTTD.........LSKCNGE
BraA.XTH9.b KDQAMGVYSSIWNADDWATQGGLVKTDWSHAPFIASYKDFK.IDACEVPTARD.........LSKCSGE
BraA.XTH5.a FNQPMKIYSSLWNADDWATRGGLEKTNWAKAPFVASYRGFH.IDGCEASVS...........AKFCETQ
BraA.XTH5.b FNQPMKIYSSLWNADDWATRGGLEKTNWAKAPFVASYRGFH.VDGCEASVN...........AKFCETQ
BraA.XTH4 FNQPMKLYSSLWNADDWATRGGLEKTNWANAPFIASYRGFH.IDGCQASVE...........AKYCATQ
BraA.XTH8 NQKPMYLFSSIWNADDWATRGGLEKTDWKKAPFVSSYKDFA.VEGCRWKDP...........FPACVST
BraA.XTH7 RFQPMGVYSTLWEADDWATRGGIEKINWSEAPFYAYYKDFD.IEGCSVPGP...........AGCPAN
BraA.XTH6 TSQPMGVYSTLWEADDWATRGGLEKIDWSKAPFYAYYKDFD.IEGCPVPGP...........TNCPSN
BraA.XTH22.c KNQPMRMYSSLWNADDWATRGGLVKTDWSKAPFTASYRGFN.QEACVWSNGKS...........SCPNG
BraA.XTH22.d KNQPMRMYSSLWNADDWATRGGLVKTDWSKAPFTASYRGFN.QEACVWSNGKS...........SCPNG
BraA.XTH22.a KNQPMRMYSSLWNADDWATRGGLVKTDWSKAPFTASYRGFN.QEACVWSNGKS...........SCPNG
BraA.XTH22.b KNRPMRMYSSLWNADDWATR......................DIIWSNGKS...........SCPDV
BraA.XTH23.a KNQPMRMYSSLWNAEDWATRGGLVKTDWSKAPFTASYRGFS.EEACVVSNGKS...........SCSNG
BraA.XTH23.b KNQPMRMYSSLWNAEDWATRGGLVKTDWSKAPFTASYRGFS.EEACVVSNGKS...........SCSNG
BraA.XTH24.a KSKPMRMYASLWNADDWATRGGLVKTDWSKAPFTASYRNIK.IDGCAHSNGRS...........SCTST
BraA.XTH24.b KSKPMRMYASLWNADDWATRGGLVKTDWSKAPFMASYRNIK.IEGCVHINGRS...........SCS..
BraA.XTH24.c KSKPMRMYASLWNADDWATRGGLVKTDWSKAPFVASYRHIK.CDGCVHANGRS...........SCSST
BraA.XTH17.e KNQPMRLYASLWEAEHWATRGGLEKTDWSKAPFTAFYRNYN.VEGCVWANGKS...........SCPG.
BraA.XTH18 KKQPMRLYASLWEAEHWATRGGLEKTDWSKAPFTAFYRNYN.VEGCVWANGKS...........SCPA.
BraA.XTH17.b KNQPMRLYASLWEAEHWATRGGLEKTDWSKAPFTAFYRNYN.VEGCVWANGKS...........SCPA.
BraA.XTH17.a KDQPMRVYASLWEAEHWATRGGLEKTDWSKAPFTAFYRNYN.VEGCVWANGKS...........SCPA.
BraA.XTH17.d KIQPMRLYASLWEAEHWATRGGLEKTDWSKAPFTAFYRNYN.VEGCVWANGKS...........SCAA.
BraA.XTH17.c KSQPMRVYASLWEAEHWATRGGLEKTDWSKAPFTAYYRNYN.VEGCVWANGKS...........SCPK.
BraA.XTH25.a KMQPMRLYSSLWNADQWATRGGLVKTDWSKAPFTASYRNFR.ADACVSYTGKP...........SCPAG
BraA.XTH25.b KLQPMRLYSSLWNADQWATRGGLVKTDWSNAPFTASYRNFR.ADACVSFAGKS...........SCPAS
BraA.XTH16 KKQPMRIYSSLWNADDWATRGGLVKTDWSKAPFTAYYRGFN.AAACTVSSGSS...........FCDPK
BraA.XTH15 KNQPMRIYSSLWNADDWATRGGLVKTDWSKAPFTAYYRGFN.AAACTVSSG..............CDPK
BraA.XTH12.a KSQPMKIYSSLWEADDWATQGGRVKTDWTNAPFSAFYRSFS.DVDCCSRTSVW...........NWVTC
BraA.XTH12.c KSQPMKIYSSLWEADDWATQGGRVKTDWTNAPFSASYRSFN.DVDCCSRTSVW...........NWVTC
BraA.XTH12.b KSQPMKIYSSLWEADDWATQGGRVKTDWSNAPFSAYYRSFS.DVDCCSRTSIW...........NWVTC
BraA.XTH14.b TSQPMKIYSSLWEADDWATQGGRVKIDWSNAPFSASYRSFN.DQSACSRTSNA...........TWVAC
BraA.XTH14.a TSQPMKIYSSLWEADDWATQGGRVKTDWSNAPFRAHYRNFD.DKNSCSRTSNL...........TWVTC
BraA.XTH22.e EKQAMRVHSSLWNGDDWATRGGLVKTDWSKAPFTAYYRNFE.VQD.CDWSAPG.........SNVSCGGG
BraA.XTH21 KSQPMRMYGSLWNADDWATRGGLVKTNWSEGPFVASFMNYN.SENACIWSIDNGTT......TNTPCSPS
BraA.XTH26 NKQGMKVFASLWSADDWATQGGRVKTNWTQAPFVAEGRRYK.ARTCLWQGPAS.........IKQCADP
BraA.XTH10 RLQPMSIKVSLWNGESWATSGGHEKVDWSKAPFVASFGDYK.IDACIWKGNPT...........LCNEE
BraA.XTH32.a .LRPMWLYGSIWDASSWATENGKYKADYKYQPFTAKYTNFK.AIGCTAYSSARCH...........PLS
BraA.XTH32.c .LRPMWLYGSIWDASSWATEDGKYKADYKYQPFSAKYTNFK.AIGCTAYSSARCH...........PVS
BraA.XTH32.b .LRPMWLYGSIWDASSWATEDGKYKADYKYQPFAAKYTNFK.AIGCTAYSPARCH...........PVS
BraA.XTH31.a .TRPMWLYGSIWDASDWATENGRIKADYRYQPFIAKYTNFK.LAGCTAEGSSSCT...........PPS
BraA.XTH31.b .TRPMWVYGSIWDASDWATENGRIKADYRYQPFVAQYRNFK.LAGCTADRSNSCR...........PPS
BraA.XTH29.a .QKPMSLYATIWDASSWATSGGKFGVDYTFSPFVSEFKDIA.LDGCNVSESLTTVTRDN..YNNINCSAS
BraA.XTH29.b .QKPMSLYATIWDASSWATSGGKFGVDYAFSPFVSEFKDVA.LDGCNVSDSFPNNNNNTVGYNYVNCSAS
BraA.XTH30 .AKPMSLYATIWDASDWATSGGKYKANYKYAPFVAEFKSFS.LDGCSVDPIQEVP.........TDCSDS
BraA.XTH27.b .SKPMSLYTTIWDGSKWATNGGKYGVNYKYAPYVAQLTDLV.LHGCAVDPIEQFP.........KCDEG
BraA.XTH27.a .SKPMSLYTTIWDGSKWATNGGKYGVNYKYAPYIARFSDLV.LHGCAVDPIEQLP.........KCDEG
BraA.XTH28 .AKPMSLYATIWDGSKWATNGGKYGVNYKFAPYVAQFTDMI.LHGCAVDPIEKVL.........SCQDG
BraA.XTH33 .SKPMSLYVTVWDGSEWATHGGKYPVNYKYGPFVASIADVK.LSGCSVNNGSSTGPGP..CTKSGGSVSS
BraA.XTH2.b PSKPMTVIASLWNGEGWATDGGKAKINWSYAPFKANFQSFS.DSGCHADGGNI...........NAEAC
BraA.XTH2.a PSKPMTLVVSLWNGEAWATEGGKAKINWSYAPFKANFERFS.DSGCHADGRTI...........NAKVC
BraA.XTH3 PSKPMQVEASLWNGDAWATDGGRTKINYAYSPFIAHFQDFSGLSGCYIDGRSD...........NVATC
BraA.XTH11.a PS...VQTMFMHG...SVKNG.SIVDPKEMPYTAEFQAST.IDGCVTDFFG..............IQKC
BraA.XTH11.b PS...VQTMFMHG...SVENG.TMVDPMEMSCTAKFQASK.IDGCATEFMS..............IDKC

PttXET16A
η6 η7 α2 β15 η8 α3
250 260 270 280 290
PttXET16A GAR.......WWDQKEFQDLDAFQYRRLSWVRQKYTIYNYCTDRSRYPS.MPPECKRDRDI........
BraA.XTH9.a DQRF......WWDEPTVSELSLHQNHQLIWVRANHMIYDYCYDAARFPV.TPLECQHHRHL........
BraA.XTH9.b EQRF......WWDEPTVSELSLHQNHQLIWVRANHMIYDYCFDAARFPV.TPLECQHHRHLIKSNYNKL
BraA.XTH5.a GKR.......WWDKEEFQDLDAKQYKRLKWVRKRYTIYNYCTDRVRFPK.PPPECRRDRDI........
BraA.XTH5.b GKR.......WWDQKEFQDLDAQQYKRLKWVRTRYTIYNYCTDRVRFPV.PPPECRRDRDI........
BraA.XTH4 GRM.......WWDQNEFRDLDAEQYRRLKWVRMKWTIYNYCTDRTRFPV.MPAECKRDRDV........
BraA.XTH8 TTEN......WWDQYDAWHLSKTQKMDYAWVQRNLVVYDYCQDRERFPK.MPWECSISPWA........
BraA.XTH7 PKN.......WWEGSAYHQLTPVEARSYRWVRVNYMIYDYCTDKSRFPV.PPPECSAGI.........
BraA.XTH6 PHN.......WWEGYAYQSLNAVEARRYRWVRVNHMVYDYCTDKSRYPV.PPVECHA...........
BraA.XTH22.c SGQGT....TGSWLS...QELDSTAQERMRWVQKNYMIYNYCTDTKRFPQGLPKECFAA.........
BraA.XTH22.d SGQGP....TGSWLS...QELDSTAQERMRWVQKNYMIYNYCTDTKRFPQGLPKECFAA.........
BraA.XTH22.a SGQGP....TGSWLS...QELDSTAQERMRWVQKNYMIYNYCTDTKRFPQGLPKECFAA.........
BraA.XTH22.b SKQGT....TGSWLS...QELDSTAQERMRWVQRNYMIYNYCTDAKRFPQGLPKECLAA.........
BraA.XTH23.a SAQGSG...SGSWLS...QELDSTGQERMRWVQSNYMIYNYCTDAKRFPQGLPRECLSA.........
BraA.XTH23.b SGQGSS...SGSWLS...QELDSTGQERMRWVQSNYMIYNYCTDAKRFPQGLPRECLSA.........
BraA.XTH24.a K....P...TSTWYT...QEMDSTSQARLRWVQKNYMIYNYCTDTKRFPQGIPRECATRS........
BraA.XTH24.b .....P...SSSWYT...QQMDATSQARLRWVQKNYMIYNYCTDRKRFPQGIPRECAPSA........
BraA.XTH24.c R....P...TSNWYT...QQMDSTSQARLRWVQKNYMIYNYCTDSKRFPQGIPRECATSA........
BraA.XTH17.e .........NSPWFT...QKVDFEGQKKMKWAQSKYMIYNYCTDKRRFPRGVPAVCT...........
BraA.XTH18 .........NAQWFT...QKVDLEGEKKMKWAQSKYMIYNYCTDKRRFPQGVPAVCT...........
BraA.XTH17.b .........NSPWFT...QTLDQRGIDKMKWAQSKYMVYNYCTDTKRFPKGVPKVCT...........
BraA.XTH17.a .........NSPWFT...QTLDKSGIDKVKWAQSKYMVYNYCTDTKRFPKGVPKVCT...........
BraA.XTH17.d .........NSPWYT...QKLDQRGMNRVKWAQRNYMVYNYCTDKKRFPKGVPAVCT...........
BraA.XTH17.c .........KSHWFT...KKLGKGGMNKVRWAQRKYMVYNYCTDKKRFPQGVPAVCT...........
BraA.XTH25.a .........SPRWLS...HRFDLTAENNMRVVQRKFMVYNYCTDSKRFPQGFPKECGIH.........
BraA.XTH25.b .........SPRWFT...QKLDLTAEDKMRVVQSKFMVYNYCTDTKRFPQGVPKECNIH.........
BraA.XTH16 FRSTFAD..GESQVP...NELNAYGRRRLRWVQKYFMIYDYCSDLKRFPQGFPPECRRSRV.........
BraA.XTH15 FKTSLSD..SELQTA...NELNAYGRRRLRWVQKYFMIYNYCSDLKRFPRGFPPECRRSRV.........
BraA.XTH12.a NAN......SNSWMW...TTLNPNQVGQMKWVQDDYMIYNYCTDYKRFPQGLPTECNLG.........
BraA.XTH12.c NAN......SNSWMW...TTLNSNQLGQMKWVQDDYMIYNYCNDLKRFPQGLPTECNLG.........
BraA.XTH12.b NAN......SNSWMW...TTLNSNQMGQMKWVQDEYLIYNYCTDYKRFPLGLPTECNLA.........
BraA.XTH14.b DAN......KDSWMW...TSLNNHQYGQMKWVQDEFMIYNYCTDYKRFPQGLAKECNL..........
BraA.XTH14.a DGN......KNSWMW...TTLNNHQIGQMNWVQKEFLIYNYCTDYKRFPQGFAKECYL..........
BraA.XTH22.e GPNS........WLN...EGIDENSRKRLKWVQSNFIIYNYCNDAKRFRLGLPTECVVMN.........
BraA.XTH21 GSSSSSTSTSEWFSQ...RGMDSSSRKVLKWVQKKFMVYNYCKDKKRFWQGLPVECGAKKNNKNKNKNKN
BraA.XTH26 TIK......SNWWTSPSFSQLTRSQIDRMQKIRSGFMIYDYCKDTNRFKGVMPPECSKRQF........
BraA.XTH10 NN.......ENWWNKNEFSSLTRVQKRWFKWVSKYHLIYDYCQDYARFHNKLPKECSLPKY........
BraA.XTH32.a ASP.........YRS...GGLTRKQYQAMRWVQTHNMVYNYCKDYKRDHSLTPECGR...........
BraA.XTH32.c ASP.........YRS...GGLTRQQYQAMRWVQTHSMVYNYCKDYKRDHSLTPECWR...........
BraA.XTH32.b ASP.........YRS...GGLTRNQYQAMRWVQTHNMIYNYCKDYKRDHSLTPECLR...........
BraA.XTH31.a ASP.........MGN...RGLSQQQMGAMAWAQRNFLVYNYCHDPKRDHTQTPEC.............
BraA.XTH31.b AAP.........TGN...RGLSQQQMGAMAWAQRNFLVYNYCHDPKRDHTQTPEC.............
BraA.XTH29.a DQL.........LMTSDYSTISPKQAAAMRRFRERYMYYSYCYDTVRYAVPPPECVILTAEKDRFRDTGR
BraA.XTH29.b DQD.........LMASDYSTISPKQAAAMRRFRERYMYYSYCYDTVRYAVPPPECVIVTAEKDRFKDTGR
BraA.XTH30 VDF.........LESQDYFSINSRQRAAMRRFRQRFMYYSYCYDTVRYPEAPPECVIVPAEKDRFKDTGR
BraA.XTH27.b ADE.........DIR.AAQEISPSQRVKMGVFRRKHMTYSYCYDRNRYKVALPECVVIPAEAQRLRVHDP
BraA.XTH27.a AIE.........YIR.AAQEISLSERAKMEVFRRKHMTYSYCYDRTRYKVSLPECVVNPAEAQRLRVYDP
BraA.XTH28 AEE.........DLR.LASEITDSQRKKMDSLRRKHMTYSYCYDRTRYKVALPECVVNTEEAKRLRVYDP
BraA.XTH33 LDP.........VDGQDFATLSKNQIAAMDWARRKLMFYSYCNDKSRYKVMPAEC..............
BraA.XTH2.b GST.....TYYWNTIQYSRLSANETTAFKNVRAKYMKYDYCSDKRRFPV.PPTECRLNQ..........
BraA.XTH2.a GST.....TYWWNTNQYSRLSANETTAFKNVRAKYMTYDYCSDHPRYPV.PPTECRLNQ..........
BraA.XTH3 GSS.....NYWWNGGKYQRLSGYEQKIYEHIRKKYMNYDYCTDRSKYQS.PPRECY............
BraA.XTH11.a FGP.....EFWWNRKENWQLTSSERKLYMNARKVYMDYDYCSDRKLYPE.VPKEFMEDQNKNVWLWSYV
BraA.XTH11.b FSL.....EYWWNRKENWELSSKERKLYMNARKVYLDYDYCLDRKRYPK.VPQECR.........SYG
1 1

PttXET16A

```
PttXET16A     ..........................................................
BraA.XTH9.a   ..........................................................
BraA.XTH9.b   EEPTDEEENDMLDLAFGLTKAARLGCQVIARPELDGVRLAIPSATRNFAVDGFVPEPH
BraA.XTH5.a   ..........................................................
BraA.XTH5.b   ..........................................................
BraA.XTH4     ..........................................................
BraA.XTH8     ..........................................................
BraA.XTH7     ..........................................................
BraA.XTH6     ..........................................................
BraA.XTH22.c  ..........................................................
BraA.XTH22.d  ..........................................................
BraA.XTH22.a  ..........................................................
BraA.XTH22.b  ..........................................................
BraA.XTH23.a  ..........................................................
BraA.XTH23.b  ..........................................................
BraA.XTH24.a  ..........................................................
BraA.XTH24.b  ..........................................................
BraA.XTH24.c  ..........................................................
BraA.XTH17.e  ..........................................................
BraA.XTH18    ..........................................................
BraA.XTH17.b  ..........................................................
BraA.XTH17.a  ..........................................................
BraA.XTH17.d  ..........................................................
BraA.XTH17.c  ..........................................................
BraA.XTH25.a  ..........................................................
BraA.XTH25.b  ..........................................................
BraA.XTH16    ..........................................................
BraA.XTH15    ..........................................................
BraA.XTH12.a  ..........................................................
BraA.XTH12.c  ..........................................................
BraA.XTH12.b  ..........................................................
BraA.XTH14.b  ..........................................................
BraA.XTH14.a  ..........................................................
BraA.XTH22.e  ..........................................................
BraA.XTH21    IKS.......................................................
BraA.XTH26    ..........................................................
BraA.XTH10    ..........................................................
BraA.XTH32.a  ..........................................................
BraA.XTH32.c  ..........................................................
BraA.XTH32.b  ..........................................................
BraA.XTH31.a  ..........................................................
BraA.XTH31.b  ..........................................................
BraA.XTH29.a  LKFGGSHTKVHRARKRRRRN..RSTPVVSAEL..........................
BraA.XTH29.b  LKFGGS....HRARKRRKRN..RSTPVVSADQ..........................
BraA.XTH30    LKFGGTEARGRRRNRRQQRPEIESDPDERRRLLK........................
BraA.XTH27.b  VTFGGIPRRHRYGKHRSRRSRVVGTESI..............................
BraA.XTH27.a  VTFGGIPRRHRSGKHRSKRSRTDGTVSI..............................
BraA.XTH28    VTFGGIPHRHRHGKHRSK.....SRQSI..............................
BraA.XTH33    ..........................................................
BraA.XTH2.b   ..........................................................
BraA.XTH2.a   ..........................................................
BraA.XTH3     ..........................................................
BraA.XTH11.a  AASKNKKLYL................................................
BraA.XTH11.b  ..........................................................
```
