# Supplementary material for: Genome-wide identification, and phylogenetic and expression profiling analyses, of XTH gene families in Brassica rapa L. and Brassica oleracea L
Source: BMC Genomics. 2020 Nov 11;21:782. doi: 10.1186/s12864-020-07153-1 (PMC7656703; doi:10.1186/s12864-020-07153-1)
Supplement: Supplementary file 5 — Additional file 5. Structure-based sequence alignment of BolXTHs and PttXET16A. Sequences were aligned using ClustalX and generated by ESPript [70]. The secondary structure elements indicated above the alignment are those of PttXET16A, Populus tremula × tremuloides XET16A (AF515607), whose structure has been experimentally determined [45]. Blue frames indicate conserved residues, white letters in red boxes indicate strict identity, and red letters in white boxes indicate similarity. The predicted α-helices and β-strands are represented by spirals and horizontal arrows, respectively. [file 12864_2020_7153_MOESM5_ESM.pdf]

```
PttXET16A                                                                            α1
                                          1         10          20          30
PttXET16A    .................................MAAAYPWTLFLGMLVMVSGTMGAALRKPVDVAFGRNY
BolC.XTH5    ..................................MGRLPLTLCLTFLVMATVTFGVPPKKAVDVPFGRNY
BolC.XTH4    ................................MTVSSTPWALVALFLMASSNVMAIPPRKAIDVPFGRNY
BolC.XTH7    .................................MVLSLFSARNAFFISLCLFAALYRPVLSKPAKFADDF
BolC.XTH8    .................................METRCSSMAAVFFFVAALMASSSTSAVPTQS.FEENF
BolC.XTH9.b  .................................MVG.....MGWFMCMMTMVCVVSCGEAAPGAKFEELY
BolC.XTH9.a  .................................MVG.....MGWFMCMMMMVCVISCGEAAPGAKFEDLY
BolC.XTH13   ........................................................MLHHVSTGSFYDNF
BolC.XTH12   ......................................MAAFATKQSMLLLSSSLLLLIGVSTGSFYDNF
BolC.XTH15   ......................................MGQSSSFTTVMVAVLLVMMFGSAYSGNFFNEF
BolC.XTH16   ......................................MGQFLNLT.VLVTVLVLTTVGTAYSGSFNEEF
BolC.XTH20   ..................................MKSSCGTRFTFLALYLFAVQ..CVYAG...SFHKDV
BolC.XTH17.b ..................................MKSSCGTRFAFLVLFLFAVQSVCVYAGTG.SFHKDV
BolC.XTH17.a ..................................MKFSCGTRFAFLVLFLIAAQSVAVYAG...SFHKDV
BolC.XTH24.b MFTHSLLIYFIKYSYYYFSSHYIYTCLPSYLFLTFLTKHKDMSSFKIFFFSALLAAVFSASAA.DFNSDV
BolC.XTH24.a .........................................MSPFKIFFFAALLAAVFSFSAA.DFNSDV
BolC.XTH24.d ......................................................................
BolC.XTH24.c .........................................MYPFKIFFFTTLLMAVFSFSDA.DFNSDV
BolC.XTH22.a .........................................MTN.NYLLPLFLSLTVISSVSA.NFQKDV
BolC.XTH23   .......................................MAMINYSTILFPLLAAIMIYSVSA.NFHQDV
BolC.XTH25   ....................................MDRSTLILSILLTLTATNTTFFSPVLAGTFDSEF
BolC.XTH21   .............MSQTQINTSATYNLKEETSPKWKTKMVSHTLLVMSLILFLGLNTFLVAHANYLNQNI
BolC.XTH22.b ......................................MGCSPILISLSILSCSALILAG...DFRKDI
BolC.XTH26   .....................................MAGLRVQTLIFVLVTALAILDRTFVEANFDKNF
BolC.XTH11.a ..................................................MLPLRAATLSSS.....SPS
BolC.XTH11.b ......................................MRGSDQKNILIVMMVVTVAATARG.....EDG
BolC.XTH2    .................................MNKREYMFSVLDFVLVLFLIATVDAQVPGGGGFDENY
BolC.XTH32.b .......................MGSSLISLLPIFHLLVLLGSSVNAYWPPSPGYWPSSKVVSLSFYKGF
BolC.XTH32.c .......................MANSLISLLPIFHLLVLLGSSVNAYWPPSPGYWPSSKVGSLNFYKGF
BolC.XTH32.a .......................MGNSLISLLTVFQFLVLLGSSVNAYWPPSPGYWPSSKVGSLSFYKGF
BolC.XTH31   .......................MAFPLILLA.....FLVLCSSGYSQRSPSPGYYPSSRVPTSPFDRQF
BolC.XTH27.a ............................METLSRFLVFMYLFSRFVSGFTLQNLP.......VTSFEESY
BolC.XTH27.b ............................METLSRFLVFMSLFSGLVSGYTLQNLP.......ITSFEESY
BolC.XTH28   ......................................MSLFTGLVSGFALQKLP.......LIQFDEGY
BolC.XTH29.a ...................................MMVMMMFVSWRCVLGLENIN.......PIFFDEGL
BolC.XTH29.b ......................................................................
BolC.XTH30.b ........................MSKLSYNLIF...FIVFLCLGLRSSAITNLN.......TLSFEESL
BolC.XTH30.a ........................MSKLSYNLIFSIIIFVFLCLGLRSSAFTNLN.......TLSFEESL
BolC.XTH33   ...............MASLKNNNMKIMWETAVVLCLCSFSLVCSHSRKFTTPNVTRLTDQFSRIAIESGF
```

```
               β1    η1   β2  η2     β3         β4          β5              TT    β6
PttXET16A
              40          50          60          70          80          90          100
PttXET16A    VPTWAFDHIKYFNG.GNEIQLHLDKYTGTGFQSKGSYLFGHFSMQMKLVPGDSAGTVTAFYLSSQN...S
BolC.XTH5    FPTWAFDHIKYLNG.GSEVHLILDKYTGTGFQSKGSYLFGHFSMHIKMVPGDSAGTVTAFYASETCY.FS
BolC.XTH4    VPTWAFDHQKQLNG.GSELQLILDKYTGTGFQSKGSYLFGHFSMHIKLPAGDTAGVVTAFYLSSTN...N
BolC.XTH7    RITWSDTHITQIDG.GRAIQLKLDPSSGCGFASKKQYLFGRVSMKIKLIPGDSAGTVAAFYMNSDT..DS
BolC.XTH8    NIMWSENHFTTSED.GQIWNLALDNDTGT.........................................
BolC.XTH9.b  RSSWAMDHCVN..D.GEVTKLKLDNSSGAGFESRSKYLFGKVSIQIKLVEGDSAGTVTAFYMSSE...GS
BolC.XTH9.a  RSSWAMDHCVN..D.GEVTKLKLDNSSGAGFESRSKYLFGKVSIQIKLVEGDSAGTVTAFYMSSE...GS
BolC.XTH13   DIPWGNGRANIFES.GQLLTCTLDKISGSGFQSKKEYLFGKIDMKMKLVAGNSAGTVTAYYLSSK...GE
BolC.XTH12   DITWGDGRANIFES.GHLLTCTLDKISGSGFQSKKEYLFGKIDMKMKLVAGNSAGTVTAYYLSSK...GE
BolC.XTH15   DLTWGDHRGNIFNG.GNMLSLSLDRVSGSGFKSKKEYLFGRIDMQLKLVAGNSAGTVTTYYLSSQ...GA
BolC.XTH16   DLTWGEHRGKIFGG.GKMLSLSLDRVSGSGFKSKKEYLFGRIDMQLKLVAGNSAGTVTAYYLSSE...GP
BolC.XTH20   NIHWGDGRGKIHDNEGKLLSLSLDKSSGSGFQ......................................
BolC.XTH17.b KIHWGDGRGKIHDNEGKLLSLSLDKSSGSGTT......................................
BolC.XTH17.a QIHWGDGRGKVRDRDGKLLSLSLDKSSGSGFQSNQEFLYGKAEVQMKLVAGNSAGTVTTFYLKAP...GT
BolC.XTH24.b NVAWGNGRGKILNN.GQLLTLTLDKSSGSGFQSKAEYLFGKIDMQIKLVPGNSAGTVTTFYLKSE...GS
BolC.XTH24.a NVAWGNGRGKILNN.GQLLTLSLDRSSGSGFQSKTEYLFGKIDMQIKLVPGNSAGTVTTFYLKSE...GS
BolC.XTH24.d ...........................................MQIKLVPGNSAGTVTTFYLKSE...GS
BolC.XTH24.c DVAWGSDHVKILNN.GQLLTLSLDKLSGSGFQSKQEYLFGQIDMQIKLVPGNSAGIVTNFYISYSTW.RS
BolC.XTH22.a EITWGDGRGQITNN.GELLTLSLDKSSGSGFQSKNEYLFGKIDMQMKLVPGNSAGTVTTLYLKSP...GT
BolC.XTH23   EITWGDGRGQITNN.GDLLTLSLDKSSGSGFQSKNEYLFGKIDMQIKLVAGNSAGTVTAYYLKSP...GS
BolC.XTH25   DITWGDGRGKVLNN.GELLTLSLDRASGSGFQSKKEYLFGKIDMQLKLVPGNSAGTVTAYYLKSK...SD
BolC.XTH21   DITWGDGRGNFQNN.GTLLNLTLDQSSGSGFQSKAEYLYGKVDMQIKLVPGNSAGTVTTFYLKSQ...GL
BolC.XTH22.b DIVWGSDKAKILEY.GQAITLSLDKASGSGFESKAQFLFGKVSMELKLVTGNSAGTVTSYYLASK...GT
BolC.XTH26   IVTWGKHHIGTTNG...NLRLVLDKSAGSAIRSKVAHLFGTVEMLIKLVPGNSAGTVAAYYMSST...GT
BolC.XTH11.a IIN........................................LGIKAPQTKSTKVTTSFYLRSK...SS
BolC.XTH11.b FVTWG...........................DNYYTTWGHPALGIKAPQTKSTKVTTSFYLRSK...SS
BolC.XTH2    VVTWGNVLKLNQGR...EVQLSMDKTAGAAFESKHKFGSGFFQMRIKLPPKDSAGVVTAFYLTSK...GD
BolC.XTH32.b RNLWGPQHQRMDHN...ALTIWLDRVSGSGFKSVKPFRSGYFGASIKLQPGYTAGVITSLYVLKR.....
BolC.XTH32.c RNLWGPQHQRMDQN...ALTIWLDRTSGSGFKSVKPFRSGYFGASIKLQPGYTAGVITSLY.........
BolC.XTH32.a KNLWGPQHQRTDQN...GLTIWLDRTSGSGFKSVKPFRSGYFGASIKLQAGYTAGVITSLYLSNQEAHPG
BolC.XTH31   RTLWGSQHQRTEQD...VITLLLDKSSGSGFKSLRSYKSGYFGASIKLQSGYTAGVDTSLYLSNNQEHPG
BolC.XTH27.a TQLFGDKNLFVHKD.GKSVRLTLDERTGSGFVSNDLYLHGLFSASIKLPSDYSAGVVVAFYMSNGDMYEK
BolC.XTH27.b TQLFGAKNLFVHKD.GKSVRLTLDERTGSGFVSNDLYLHGFFSASIKLPSDYSAGVVVAFYMSNGDT...
BolC.XTH28   THLFGDQNMIVNRD.GKSVRLILDERTGSGFVSNDIYLHGFFSSSIKLPADYSAGVVIAFYLSNGDIYEK
BolC.XTH29.a SHLFGEANLIRSPD.DRSVRLLLDKYTGSGFISSNMYQHGFFSSLIKLPGAYTAGLVVAFYFMT......
BolC.XTH29.b ......................................................................
BolC.XTH30.b SPLFGDGNLVRSPD.DLSVRLLLDKYTGSGFISSNMYQHGFYSSMIKLPADYTAGVVVAFYTSNGDVFEK
BolC.XTH30.a SPLFGDANLVRSPD.DLSVRLLLDKYTGSGFISSNMYQHGFYSSMIKLPADYTAGVVVAFYTSNGDVFQK
BolC.XTH33   SKRFGDHNIIIN...DSLAKLTLDKSSGAGLVTKNTYHYGFFSARLKLPAGFASGVVVAFYLSNAESYPK
```

```
PttXET16A        β7 (arrow)  TT  β8 (arrow)  TT   β9 (arrow)   β10 (arrow)   β11 (arrow) TT
                   110       120       130       140       150       160
PttXET16A    EHDEIDFEFLGNRTGQPYILQTNVFTGGKGD.....REQRIYLWFDPTKEFHYYSVLWNMYMIVFLVDDV
BolC.XTH5    VHDEIDFEFLGNRTGQPYILQTNVFTGGKGN.....REQRINLWFDPSKDYHSYSVLWNMYQIVFFVDDV
BolC.XTH4    EHDEIDFEFLGNRTGQPVILQTNVFTGGKGN.....REQRIYLWFDPSKAYHTYSVLWNLYQIVFFVDNI
BolC.XTH7    VRDELDFEFLGNRSGQPYTVQTNVFAHGKGD.....REQRVNLWFDPSRDFHEYAISWNHLRIVFYVDNV
BolC.XTH8    ................................................................FFVDRV
BolC.XTH9.b  NHNEFDFEFIDNPTGEPYIVQTNVYVNGVGN.....REQRLNLWFDPTTEFHTYSILWSKRSVVFMVDET
BolC.XTH9.a  NHNEFDFEFLGNTTGEPYIVQTNVYLNGVGN.....REQRLNLWFDPTTDFHTYSILWSKRSVVFMVDET
BolC.XTH13   TWDEIDFEFLGNVTGQPYVLHTNVFTGGKGN.....REMQFYLWFDPTVDSHTYTVLWNPLNIIFIVDGI
BolC.XTH12   TWDEIDFEFLGNVTGQPYVLHTNVFTGGKGN.....REMQFYLWFDPTADFHTYTVLWNPLNIIFLVDGI
BolC.XTH15   THDEIDFEFLGNETGKPYVLHTNVFAQGKGN.....REQQFYLWFDPTKNFHTYSIVWRPQHIIFLVDNL
BolC.XTH16   THDEIDFEFLGNETGKPYVLHTNVFAQGKGN.....REQQFYLWFDPTKNFHTYSLVWRPQHIIFMVDNV
BolC.XTH20   .SNQ...EFL..............YGK...........................................
BolC.XTH17.b .WDEIDFEFLGNISGHPYTLHTNVYTKGSGD.....KEQQFHLWFDPTREFHTYCITWNPERIIFTVDNV
BolC.XTH17.a MWDEIDFEFLGNISGHPYTLHTNVYTQGSGD.....KEQQFHLWFDPTRNFHTYCITWNPQRIIFTVDGI
BolC.XTH24.b TWDEIDFEFLGNMSGDPYTLHTNVYTQGKGD.....KEQQFHLWFDPAANFHTYSILWNPQRIIWTVDNT
BolC.XTH24.a TWDEIDFEFLGNMSGDPYTLHTNVYTQGKGD.....KEQQFYLWFDPTANFHTYSILWNPQRIILTVDDT
BolC.XTH24.d TWDEIDFEFLGNMTGDPYTLHTNVYTQGKGD.....KEQQFHLWFDPTANFHTYSILWNPQRIILTVDDT
BolC.XTH24.c TWDEIGFEFLGNTSGDPYTLHTNVYTQGKGD.....KEQQFYLWFDPTANFHTYSILWNPQRIILTVDDT
BolC.XTH22.a TWDEIDFEFLGNLSGDPYTLHTNVYTQGKGD.....KEQQFKLWFDPTADFHTYTILWNPQRIIFTVDGT
BolC.XTH23   TWDEIDFEFLGNLSGDPYTLHTNVFTQGKGD.....REQQFKLWFDPTIDFHTYSILWNPQRIIFSVDGT
BolC.XTH25   TWDEIDFEFLGNLTGDPYVMHTNVYTQGKGD.....REQQFNLWFDPTADFHTYSVLWNPHHIVFLVDGI
BolC.XTH21   TWDEIDFEFLGNVSGDPYILHTNVYTQGKGD.....REQQFYLWFDPTAEFHNYSILWNPSHIVFYVDGK
BolC.XTH22.b MWDEIDFEFLGNLSGDPYTVHTNVITQGKGD.....REQQFRLWFDPTVDFHTYSILWNPKTIVFYVDGT
BolC.XTH26   AHDEIDFEFLGNSTGEPYTIHTNIFAKGKGD.....REQQFKPWFNPTNGFHNYTIHWNPSEVVWFVDGT
BolC.XTH11.a RNDGLCFQIFGN..GPAYLLNTIIFVYGEGD.....KYQRFRLWFDPTKDYHSYRFYV.........DDT
BolC.XTH11.b RNDGLCFQIFGN..GPAYLLNTIIFVYGEGD.....KYQRFRLWFDPTKDYHSY..............SDP
BolC.XTH2    THDEVDFEFLGNRDGKPIQIQTNVFNNGQGN.....REQKFVLWFDPSTDFHTYGILWNPYQIVFYVDKI
BolC.XTH32.b ...................................................ESYELYFPGLGPRFLVDDI
BolC.XTH32.c .....................................................YDSFTP...MRFLVDDI
BolC.XTH32.a FHDEVDIEFLGTTSGKPYTLQTNVYIRGSGDGKIIGREMKFRLWFDPTSGFHHYAILWNPREIIFLVDDI
BolC.XTH31   DHDEVDIEFLGTTPGKPYSLQTNVFVRGSGDRNVIGREMKFNLWFDPTQDFHHYAILWNPNQIVFYVDDV
BolC.XTH27.a NHDEIDFEFLGNIRGKEWRIQTNIYGNGSTHL...GREERSNLWFDPTEDYHQYSILWSDSHIIFFVDNI
BolC.XTH27.b ................................................................FYVDNV
BolC.XTH28   NHDEIDFEFLGNIRGKEWRIQTNIYGNGSTHL...GREERYNLWFDPTEDFHQYSIMWSHSHIIFYVDNI
BolC.XTH29.a ..............................................................CRFWVDDV
BolC.XTH29.b ......................................................................
BolC.XTH30.b THDELDIEFLGNIKGKPWRFQTNLYGNGSTHR...GREERYRLWFDPSKEFHRYSILWTPHKIIFWVDDV
BolC.XTH30.a THDELDIEFLGNIKGKPWRFQTNLYGNGSTPR...GREERYRLWFDPSKEFHRYSILWTPHKIIFWVDDV
BolC.XTH33   NHDEIDIELLGRSRRDDWSIQTNVYANGSLKT...GREEKFYYWFDPTQAFHDYTLIWNSHHIVFLVDNI
```

```
PttXET16A    β12 (arrow)  η3            β13 (arrow)       η4      η5     β14 (arrow) .  (arrow) T
             170       180       190       200       210       220       230
PttXET16A    PIRVFKNCKDLGVK....FPFNQPMKIYSSLWNADDWATRGGLEKTDWSKAPFIASYRSFH.IDGCEASV
BolC.XTH5    PIRVFKNSKDIGVK....FPFNQPMKIYSSLWNADDWATRGGLEKTNWAKAPFVASYRGFH.VDGCEASV
BolC.XTH4    PIRVFKNAKDLGVR....FPFNQPMKLYSSLWNADDWATRGGLEKTNWANAPFIASYRGFH.IDGCQASV
BolC.XTH7    PIRVYKNNEARKVP....YPRFQPMGVYSTLWEADDWATRGGIEKINWSEAPFYAYYKDFD.IEGCSVPG
BolC.XTH8    PIRVYKN..SDKVPNNDFFPNQKPMYLFSSIWNADDWATRGGLEKTDWKKAPFVSSYKDFA.VEGCRWKD
BolC.XTH9.b  PIRVHNNLEDKGIP....FAKDQAMGVYSSIWNADDWATQGGLVKTDWSHAPFIASYKDFK.IDACEVPT
BolC.XTH9.a  PIRVHKNLEDKGIP....FAKDQAMGVYSSIWNADDWATQGGLVKTDWSHAPFIASYKDFK.IDACEVPT
BolC.XTH13   PIRMFKNNEAHGVA....CLKSQRMKIYSSIWEADDWATQGGRVKTDWTNAPFSAFYRSFN.DVDCCSRT
BolC.XTH12   PIRVFKNNEAHGVA....YPKSQPMKIYSSLWEADDWATQGGRVKTDWTNAPFSASYRSFN.DVDCCSRT
BolC.XTH15   PIRVFNNAEKLGVP....FPKNQPMRIYSSLWNADDWATRGGLVKTDWSKAPFTAYYRGFN.AAACTVSS
BolC.XTH16   PIRVFNNAEQLGVP....FPKKQPMRIYSSLWNADDWATRGGLVKTDWSKAPFIAYYRGFN.AAACTVSS
BolC.XTH20   ..AEFKNSESIGIH....FPKSQPMRVYASLWEAEHWATRGGLEKTDWSKAPFTAYYRNYN.VEGCVWAN
BolC.XTH17.b PIREFKNSESIGIP....FPKIQPMRLYASLWEAEHWATRGGLEKTDWSKAPFTAFYRNYD.VEGCVWAN
BolC.XTH17.a PIREFKNSESIGVP....FPKNQPMRLYASLWEAEHWATRGGLEKTDWSKAPFTAFYRNYN.VEGCVWAN
BolC.XTH24.b PIREFKNSEPLGVL....FPKSKPMRMYASLWNADDWATRGGLVKTDWSKAPFVASYRHIK.CDGCVHAN
BolC.XTH24.a PIREFKNSESIGVL....FPKSKPMRMYASLWNADDWATRGGLVKTDWSKAPFMAAYRNIK.IDGCAHIN
BolC.XTH24.d PIREFKNSESLGVL....FPKSKPMRMYASLWNADDWATRGGLVKTDWSKAPFTASYRNIK.VDGCAHSN
BolC.XTH24.c PIREFKNSESIGVL....FPNSQPMRMYANLWNADDWATRGGRVKTDWSGAPFTASFRNIN.IDGCVHTN
BolC.XTH22.a PIREFKNMESIGTL....FPKNRPMRMYSSLWNADDWATRGGLVKTDWSKAPFTASYRGFN.QEACVWSN
BolC.XTH23   PIREFKNMESQGTL....FPKNQPMRMYSSLWNAEDWATRGGLVKTDWSKAPFTASYRGFS.EEACVVSN
BolC.XTH25   AIRQFKNLEHRGIQ....YPKLQPMRLYSSLWNADQWATRGGLVKTDWSNAPFTASYRNFR.ADACVSFA
BolC.XTH21   PIREFKNLEAMGVA....YPKSQPMRMYGSLWNADDWATRGGLVKTNWSEGPFVASFMNYNSENACVWST
BolC.XTH22.b PIREFKKMNTKNVA....YPEKQAMRVHSSLWNGDDWATRGGLVKTDWSKAPFTAYYRNFE.VQDCDWSA
BolC.XTH26   PIRVFRNYEKEGIA....YPNKQGMKVFASLWSADDWATQGGRVKTNWTQAPFVAEGRKYK.ARACLWQG
BolC.XTH11.a AIRVYKKNPDI.YYPSV.....QTMFMHGSVENR.........TMVDPKEMSYTAKFQASK.IDGCATEF
BolC.XTH11.b GVQEESR.....HLLPI.....GADDVH.AVENG.........TMVDPKEMSYTAKFQASK.IDGCATEF
BolC.XTH2    PIRIFKNNKKYGVGYPS.....KPMTVIASLWNGEGWATDGGKAKINWSYAPFKANFQSFS.DSGCHADG
BolC.XTH32.b PIRRYPKKSAATFP.......LRPMWLYGSIWDASSWATEDGKYKADYKYQPFSAKYTNFK.AIGCTAYS
BolC.XTH32.c PIRRYPKKSAATFP.......LRPMWLYGSIWDASSWATENGKYKADYKYQPFTAKYTNFK.AIGCTAYS
BolC.XTH32.a PIRRYPKKSAATFP.......LRPMWLYGSIWDASSWATEDGKYKADYKYQPFTAKYTNFK.AIGCTAYS
BolC.XTH31   PIRTYDRKNEAIFP.......TRPMWLYGSIWDASDWATENGRIKADYRYQPFFAKYTNFK.LAGCTTEG
BolC.XTH27.a PIREVKRTASMGGHFP.....SKPMSLYTTIWDGSKWATNGGKYGVNYKYAPYVAKLTDLV.LHGCAVDP
BolC.XTH27.b PIREVKRTSSMGGDFP.....SKPMSLYTTIWDGSKWATNGGKYGVNYKYGPYIARFSDLV.LHGCAVDP
BolC.XTH28   PIREVKRTASMGGDFP.....AKPMSLYATIWDGSKWATNGGKYGVNYKFAPYVAQFADLI.LHGCAVDP
BolC.XTH29.a PIREIIRKEEMKGDYP.....QKPMSLYATIWDASSWATSGGKFGVDYAFSPFVSEFKDVA.LDGCNVSD
BolC.XTH29.b ..........MNGDYP.....QKPMSLYATIWDASSWATSGGKFSVDYTFSPFVSEFKDIA.LDGCNVSE
BolC.XTH30.b PIREVIRSEAMGADYP.....AKPMSLYATIWDASDWATSGGKYKANYKYAPFVAEFKSFS.LDGCSVDP
BolC.XTH30.a PIREVIRSEAMGADYP.....AKPMALYATIWDASDWATSGGKYKANYKFAPFVAEFKSFS.LDGCSVDP
BolC.XTH33   PVREFPNRGAFMSAYP.....SKPMSLYVTVWDGSEWATHGGKYPVNYKYGPFMASIADVK.LSGCSVNN
```

```
                            η6   η7          α2           β15  η8
PttXET16A   T.............T
                     240          250        260          270        280

PttXET16A   E.............AKFCATQ......GARWWDQKEFQDLDAFQYRRLSWVRQK...YTIYNYCTDRSRY
BolC.XTH5   N.............AKFCETQ......GKRWWDQKEFQDLDAQQYKRLKWVRTR...YTIYNYCTDRVRF
BolC.XTH4   E.............AKYCATQ......GRMWWDQNEFRDLDAEQYRRLKWVRMK...WTIYNYCTDRTRF
BolC.XTH7   ..............PAGCPAN......PKNWWEGSAYHQLTPVEARSYRWVRVN...HMIYDYCTDKSRF
BolC.XTH8   P............FPACVSTT......TENWWDQYDAWHLSKTQKMDYAWVQRN...LVVYDYCKDRERF
BolC.XTH9.b TA..........DLSKCSGEE......QRFWWDEPTVSELSLHQNHQLIWVRAN...HMIYDYCFDAARF
BolC.XTH9.a TT..........DLSKCNGED......QRFWWDEPTVSELSLHQNHQLIWVRAN...HMIYDYCYDAARF
BolC.XTH13  S..........VWNWVTCNA.......NSNSWMWTT...LNPNQVGQMKWVQDD...YMIYNYCTDYKRF
BolC.XTH12  S..........VWNWVTCNA.......NSNSWMWTT...LNSNQLGQMKWVQDD...YMIYNYCNDFKRF
BolC.XTH15  G.........CDPKFKTSLS.......DSELQTANE...LNAYGRRRLRWVQKY...FMIYNYCSDLKRF
BolC.XTH16  GSSF......CDPKFRSTFA.......DGESQVPNE...LNAYGRRRLRWVQKY...FMIYDYCSDLKRF
BolC.XTH20  .............GKSSCPK.......KSHWFTKK....LGKGGMNKVKWAQRK...YMVYNYCTDKKRF
BolC.XTH17.b .............GKSSCAA.......NSPWYTQK....LDQRGMNRVKWAQRN...YMVYNYCTDKKRF
BolC.XTH17.a .............GKSSCPA.......NSPWFTQT....LDQRGIDKMKWAQSK...YMVYNYCTDTKRF
BolC.XTH24.b .............GRSSCSS....TRPTSNWYTQQ....MDSTSQARLRWVQKN...YMIYNYCTDSKRF
BolC.XTH24.a .............GRSSCS.......PSSSWYTQQ....MDATSQARLRWVQKN...YMIYNYCTDRKRF
BolC.XTH24.d .............GRSSCTS....TKPTSNWYTQE....MDSTSQARLRWVQKN...YMIYNYCTDTKRF
BolC.XTH24.c .............GRSSCST....NKPSSSWYTQQ....MDATSQARLEWVQKN...YMIYTYCTDTEKF
BolC.XTH22.a .............GKSSCPDVSKQGT.TGSWLSQE....LDSTAQERMRWVQRN...YMIYNYCTDAKRF
BolC.XTH23  .............GKSSCSNGSGQGSGSGSWLSQE....LDSTGQERMRWVQSN...YVIYNYCTDAERF
BolC.XTH25  .............GKSSCPAS......SPRWFTQK....LDLTAEDKMRVVQSK...FMVYNYCTDTKRF
BolC.XTH21  DNGTTTTTP.CSPGDSSSSSS....TSTSEWFSQRG...MDSSSRKVLKWVQKK...FMVYNYCKDKKRF
BolC.XTH22.b PG...........SNASCGGG.....GPNSWLNEG....IDENSRKRLKWVESN...FIIYNYCNDAKRF
BolC.XTH26  P...........VSIKQCADP....TIKSNWWTSPS...FSQLTRTQLDRMQKIRSGFMIYDYCKDTNRF
BolC.XTH11.a MS...........IDKFFG........LEYWWNRKENWELSSKERKLYMNARKV...YLDYDYCLDRKRY
BolC.XTH11.b MS...........IDKFFG........LEYWWNRKENWELSSKERKLYMNARKV...YLDYDYCLDRKRY
BolC.XTH2   GN...........INAEACGS......TTYYWNTIQYSRLSANETTAFKNVRAK...YVTYDYCSDKRRF
BolC.XTH32.b S.............ARCHPVS......ASPYRSGG....LTRQQYQAMRWVQTH...SMVYNYCKDYKRD
BolC.XTH32.c S.............ARCHPLS......ASPYRSGG....LTRKQYQAMRWVQTH...NMVYNYCKDYKRD
BolC.XTH32.a P.............ARCHPVS......ASPYRSGG....LTRKQYQAMRWVQTH...NMIYNYCKDYKRD
BolC.XTH31  S.............SSCTPPS......ASPMGNRG....LSQQQMGAMAWAQRN...FLVYNYCHDPKRD
BolC.XTH27.a IEQFP...........KCDEG.......ADEDIR.AAQEISPSQRVKMGAFRRK...HMTYSYCYDRNRY
BolC.XTH27.b IEQFP...........KCEER.......AIEYIR.AAQEISPSERAKMEVFRRK...HMTYSYCYDRTRY
BolC.XTH28  IEKVL...........SCQDG.......AAEDLR.LASEITESQRKKMDSLRRK...HMTYSYCYDRTRY
BolC.XTH29.a SFPNNNNNNTVGYNYINCSAS.......DQDLMTSDYSTISPKQAAAMRRFRER...YMYYSYCYDTVRY
BolC.XTH29.b SLTTLTRDN...YNNINCSAS.......DQLLMTSDYSTISPKQAAAMRRFRER...YMYYSYCYDTVRY
BolC.XTH30.b IQEVP..........TDCSDS.......VDFLESQDYFSINSRQRASMRRFRQR...FMYYSYCYDTVRY
BolC.XTH30.a IQEVP..........VDCADS.......VEFLESQDYSAINSHQRAAMRRFRQK...FMYYSYCYDTVRY
BolC.XTH33  GSSTGPGPCTK...SGGSVSS.......LDPVDGQDFATLSKNQIAAMDWARRK...LMFYSYCNDKSRY
                                                                       1
```

```
                 α3
PttXET16A
                 290

PttXET16A   PS.MPPECKRDRDI.................................................
BolC.XTH5   PV.PPPECRRDRDI.................................................
BolC.XTH4   PV.MPAECKRDRDV.................................................
BolC.XTH7   PV.PPPECSAGI...................................................
BolC.XTH8   PK.MPWECSISPWA.................................................
BolC.XTH9.b PV.TPLECQHHRHL.................................................
BolC.XTH9.a PV.TPLECQHHRHL.................................................
BolC.XTH13  PQGLPMECNLG....................................................
BolC.XTH12  PQGLPTECNLG....................................................
BolC.XTH15  PRGFPPECRRSRV..................................................
BolC.XTH16  PQGFPPECRRSRV..................................................
BolC.XTH20  PQGVPAVCT......................................................
BolC.XTH17.b PKGVPAVCT......................................................
BolC.XTH17.a PKGVPKVCT......................................................
BolC.XTH24.b PQGIPRECATSA...................................................
BolC.XTH24.a PQGIPRECATSA...................................................
BolC.XTH24.d PQGIPRECATRS...................................................
BolC.XTH24.c PQGVPSECATSV...................................................
BolC.XTH22.a PEGLPKECLAA....................................................
BolC.XTH23  PQGLPRECLSA....................................................
BolC.XTH25  PQGVPKECNIH....................................................
BolC.XTH21  WQGLPVECDAKKNNKNKNKNKNKNIKS....................................
BolC.XTH22.b RLGLPTECVVMN...................................................
BolC.XTH26  KGVIPPECSKRQF..................................................
BolC.XTH11.a PK.VPQECRSYG...................................................
BolC.XTH11.b PK.VPQECRSYG...................................................
BolC.XTH2   PV.PPTECRLNQ...................................................
BolC.XTH32.b HSLTPECWR......................................................
BolC.XTH32.c HSLTPECGR......................................................
BolC.XTH32.a HSLTPECLR......................................................
BolC.XTH31  HTQTPEC........................................................
BolC.XTH27.a KVALPECVVIPAEAQRLRVHDPVTFGGIPRRHRNGKHRSRRSRVVGAESMLLEQSRYNETYSQ
BolC.XTH27.b KVSLPDCVVNPAEAHRLRVYDPVTFGGIPRRHRSGKHRSKRSHMDGTESI.............
BolC.XTH28  KVALPECVVNPEEAKRLRVYDPVTFGGIPHRHGHGKHRSK.....SRQSI.............
BolC.XTH29.a AVPPPECVIVTAEKDRFRDTGRLKFGGS....HRARKRRKRN..RSTPVVSADQ.........
BolC.XTH29.b AVPPPECVILTAEKDRFRDTGRLKFGGSHTKVHRARKKRRRN..RSTPVVSAEL.........
BolC.XTH30.b PEAPPECVIVPAEKDRFKDTGRLKFGGTEARGRRRNHRQQRPEIESDPDERRRLLK.......
BolC.XTH30.a PEPPPECVIVPAEKDRFRDTGRLKFGGTEARERRRNRRQQRPEIESDPDERKLL.........
BolC.XTH33  KVMPAEC........................................................
                  1
```
